# Tissue-Specific Phytochemical Profiles of Two Huyou Cultivars: Bioactive Compounds and Thermal Processing Effects

**DOI:** 10.3390/foods15213805

**Published:** 2026-10-23

**Authors:** Mei Lin, Weiqing Zhang, Zhoulin Yao, Tianyu Wang, Youming Shen, Xianju Feng, Mingxia Wen, Wei Li, Bei Huang

**Affiliations:** 1 Institute of Citrus Research, Zhejiang Academy of Agricultural Sciences, Taizhou 318026, China; 2 Research Institute of Pomology, Chinese Academy of Agricultural Sciences, Xingcheng 125100, China

**Keywords:** Huyou (*Citrus changshanensis*), cultivar comparison, thermal processing, metabolite compartmentalization, bioactive compounds

## Abstract

(1) Background: Huyou (*Citrus changshanensis*) is a traditional Chinese medicinal and edible citrus, yet cultivar- and tissue-dependent variations in metabolite profiles and their differential responses to thermal processing remain poorly characterized. (2) Methods: Physicochemical properties, targeted metabolite profiles (sugars, organic acids, amino acids, flavonoids, phenolic acids, limonoids), and antioxidant capacities were compared across five tissues (flavedo, albedo, segment membranes, pulp, seeds) of two Huyou cultivars, ‘Changhu No. 1’ (HY-N1) and ‘Huyou 01-7’ (HY-17), under freeze-drying and hot-air drying (60 °C). (3) Results: HY-N1 pulp had higher soluble sugars, acidity, and amino acids but lower juice yield and greater limonoid bitterness; HY-17 exhibited a more balanced sugar-acid profile and reduced bitterness, offering metabolic clues for its prospective fresh-consumption potential. Tissue-specific distribution revealed flavonoids concentrated in peel and segment membranes, while phenolic acids and limonoids accumulated in flavedo and seeds. Across tissues, HY-N1 maintained higher levels of most flavonoids and phenolic acids than HY-17. Hot-air drying degraded heat-labile amino acids and flavanone glycosides, yet enriched polymethoxyflavones and phenolic acids presumably through matrix-associated effects. Antioxidant capacity aligned spatially with metabolite allocation and showed variable responses to hot-air drying. (4) Conclusions: These findings inform cultivar-specific utilization, tissue-selective valorization, and thermal processing strategies of Huyou.

## 1. Introduction

Citrus fruits are among the most economically important fruit crops worldwide, valued for their pleasant flavor and health-promoting nutrients, including vitamin C, flavonoids, and dietary fiber [1,2]. Beyond fresh consumption, drying is a key processing strategy [3]. In China, dried citrus fruits and peels, such as Zhishi and Chenpi (dried mandarin peel), are critical ingredients in functional foods and herbal medicine [4]. Hot-air drying is the most widely used industrial technique for these products; however, the nutritional and functional quality of dried products is highly dependent on the inherent composition of the raw material and the thermal stability of specific bioactive compounds.

**Figure 1:**
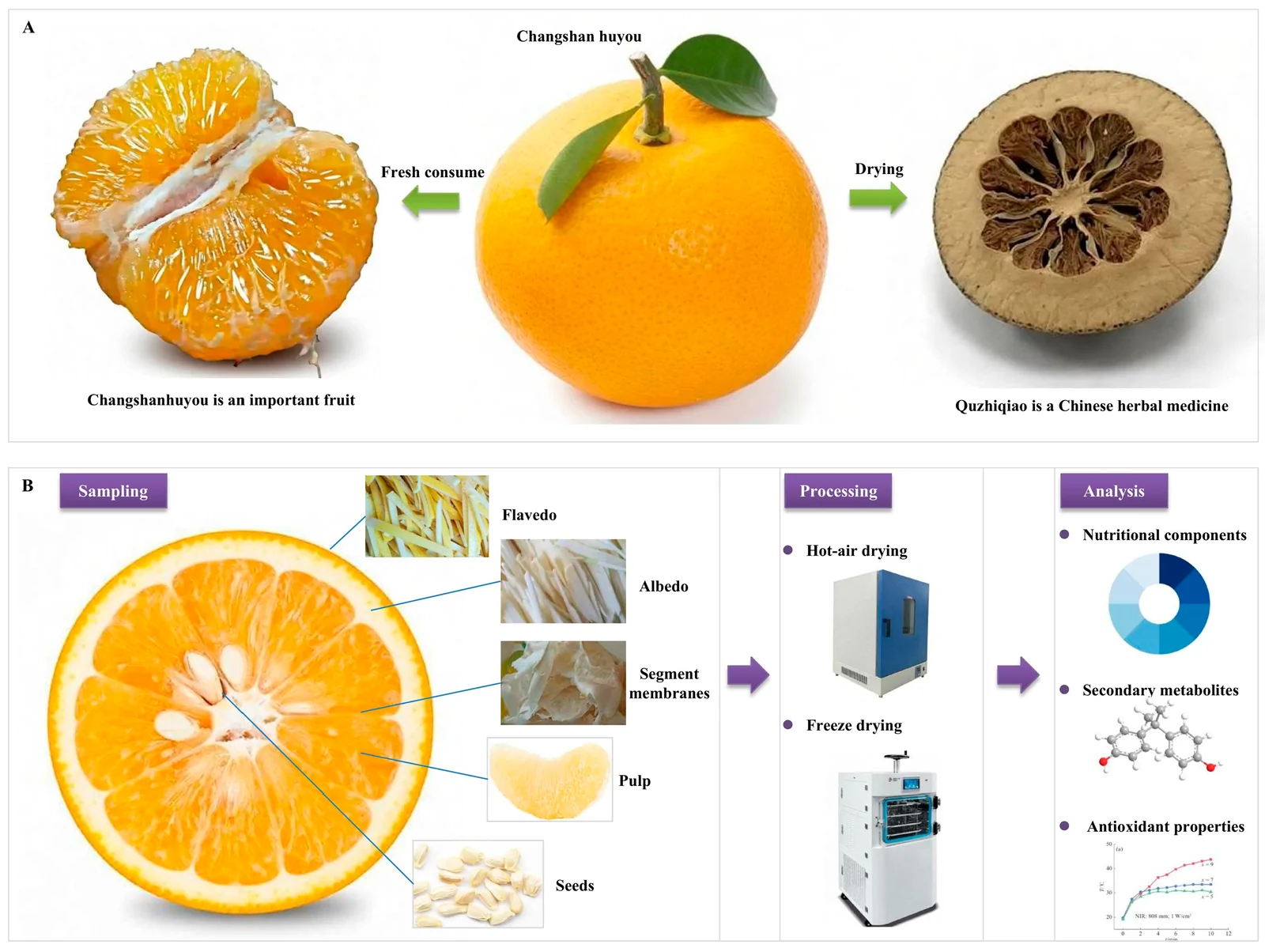
Schematic diagram of the experimental design. (**A**) Huyou fruits were separated into five fruit tissues and subjected to freeze-drying or hot-air drying. (**B**) Samples were analyzed for nutritional components, secondary metabolites, and antioxidant properties.

Huyou (commonly referred to as *Citrus changshanensis* or Changshan Huyou) is a distinctive pomelo-derived citrus cultivar native to Zhejiang Province, China (Figure 1A) [4]. Genetic evidence suggests it originated from a natural hybridization involving *Citrus grandis* [5], with a documented cultivation history of over 600 years in the Changshan region. It holds the designation of China National Geographical Indication Product, a status that reflects its unique germplasm value. In 2023, Huyou cultivation covered over 7000 ha in Changshan County, yielding 142,000 tonnes of fruit. This output generated a total industrial value of 4.72 billion yuan, highlighting the crop’s pivotal socioeconomic role in the region [6]. Beyond fresh consumption, Huyou is extensively processed into dried products. Most notably, Qu Zhi Qiao, a traditional medicinal and edible commodity, is valued for its efficacy in relieving respiratory and gastrointestinal discomforts [7]. Despite its established agroeconomic importance, the phytochemical composition of Huyou remains poorly defined at the tissue-level.

While previous studies have characterized the nutritional and functional components of Huyou, significant gaps remain regarding inter-cultivar variation and changes in bioactive compounds during processing [8,9]. The Huyou fruit has a complex anatomical architecture comprising five distinct tissues—flavedo, albedo, segment membranes, pulp, and seeds—each showing a distinct distribution of nutrients and bioactive compounds [8]. Earlier studies have characterized Huyou germplasm quality and the presence of flavonoids and limonoids in the peel; however, tissue-specific metabolic profiling across cultivars remains poorly characterized. Moreover, drying is a critical processing step for Huyou, as the chosen drying regime directly determines final product quality and phytochemical profiles. Several investigations have explored drying-mediated phytochemical alterations in citrus fruits and Huyou-derived by-products, though with important limitations [10,11,12]. For example, drying-induced compound losses were documented in physiologically dropped immature citrus fruits and navel orange peel, yet those works focused on single tissue or non-mature materials [10,12]. One Huyou-related study addressed processing waste but mainly targeted dietary fiber properties rather than comprehensive metabolite responses [11]. Freeze-drying is widely recognized for its ability to retain nutrients, yet hot-air drying remains the industrial standard. The thermal lability of key bioactive compounds poses a fundamental challenge: elevated temperatures can degrade heat-sensitive constituents, thereby compromising the fruit’s functional efficacy [10,13]. Thus, understanding how specific drying conditions affect metabolite stability is essential for process optimization.

In this study, we systematically mapped the tissue-specific metabolome of two dominant Huyou cultivars, ‘Changhu No 1’ (HY-N1) and ‘Huyou 01-7’ (HY-17), across five anatomically distinct tissues. We hypothesized that (i) HY-N1 and HY-17 exhibit divergent tissue-level distribution patterns for primary and secondary metabolites; (ii) hydrophilic flavanone glycosides and lipophilic polymethoxyflavones may differ in thermal stability, possibly linked to matrix-related properties; and (iii) hot-air drying modulates antioxidant capacity in a tissue- and assay-dependent manner. Using freeze-drying as a thermally inert reference, we evaluated the fate of key bioactive compounds under hot-air drying. This work maps the phytochemical basis of Huyou quality formation and identifies distinct cultivar-specific patterns in tissue-level metabolite distribution, providing a metabolite-guided framework for targeted cultivar selection, processing optimization, and high-value by-product utilization.

## 2. Materials and Methods

### 2.1. Sample Collection and Preparation

Two major Huyou cultivars, HY-N1 and HY-17, were used in this study. Fruits were harvested from the standardized cultivation orchard of the Zhejiang Citrus Research Institute, located in Aijia Dagongshan, Changshan County, Zhejiang Province, China (118°27′22.55″ E, 28°53′45.33″ N), in late 2024. All trees were approximately 30 years old and at full fruit-bearing maturity. For each cultivar, fifteen uniformly growing trees were randomly selected, and five commercially mature fruits were collected per tree, yielding a total of 75 fruits for each cultivar. To form three independent biological replicates, the 75 fruits per cultivar were randomly assigned into three groups containing 25 fruits per group.

Intact fruits were promptly subjected to physical trait analysis, including fruit weight, dimensions, peel color, edible rate, and juice yield (Section 2.2) [14,15]. Following physical measurements, each fruit was anatomically dissected into five distinct tissues: flavedo, albedo, segment membranes, pulp, and seeds (Figure 1B). The pulp was mechanically juiced using a domestic juicer, and the extracted juice was immediately analyzed for soluble solids content (SSC), titratable acidity (TA), and vitamin C levels (Section 2.2).

For phytochemical profiling, tissues from fruits within the same biological replicate were pooled and homogenized separately by tissue type. Each tissue homogenate was then split into two equal aliquots for two drying treatments: (i) Vacuum freeze-drying (FD): samples were spread at a thickness of 8–10 mm and lyophilized using a CHRIST Alpha 1-2 LD plus freeze dryer. Following pre-freezing to −30 °C, vacuum was applied. Primary drying was operated at 0.05–0.2 mbar, with sample temperature maintained 2–5 °C below the eutectic point; secondary drying was performed at ~0.01 mbar, with sample temperature raised to 20 °C and the cold trap set at −55 °C. The pressure rise test was used to confirm the drying endpoint, and the process was terminated once a constant weight was attained; (ii) Hot-air drying (HAD): samples were dried at 60 °C using a hot air dryer (JINHONG DHG-9140A, Shanghai, China) until a constant weight was attained. All dried samples were ground into a homogeneous fine powder (40 mesh) using a stainless-steel grinder, then hermetically sealed in opaque, light-proof tubes and stored at −80 °C pending analysis of flavonoids, phenolic acids, limonoids, and antioxidant capacity (Section 2.3, Section 2.4, Section 2.5 and Section 2.6).

### 2.2. Determination of Fruit Appearance and Physical Traits

Agronomic traits were assessed following established protocols for citrus fruit evaluation [16]. Individual fruit weight was recorded to the nearest 0.01 g using an electronic analytical balance. Transverse and longitudinal diameters were measured with digital calipers (0.01 mm precision), and the fruit shape index was computed as the ratio of longitudinal to transverse diameter. Peel thickness was determined at the equatorial plane of the fruit cross-section. Edible rate was calculated based on the proportion of peeled pulp weight to total fruit weight, while juice yield was quantified as the percentage of filtered juice weight relative to the original pulp weight.

External peel color was measured using a Chroma Meter CR-400 (Konica Minolta, Tokyo, Japan) in the CIELAB color space. Prior to measurement, the instrument was calibrated against standard white and black calibration tiles. For each fruit, color parameters were recorded at three equidistant points around the equatorial zone. The mean values of lightness (L*), red–green coordinate (a*), yellow–blue coordinate (b*), and total color difference (ΔE*ab) were calculated and used for subsequent statistical analysis [17].

Soluble solids content was determined with a PAL-1 refractometer (Atago, Tokyo, Japan). Total sugar content was assessed via the 3,5-dinitrosalicylic acid (DNS) colorimetric method, and titratable acid was quantified by potentiometric titration [18]. Vitamin C content was determined by the 2,6-dichloroindophenol (DCPIP) titration method [18].

### 2.3. Determination of Soluble Sugars, Organic Acids, and Free Amino Acids

Soluble sugars (sucrose, glucose, fructose), organic acids (tartaric, malic, shikimic, citric, quinic, lactic, acetic, succinic, and fumaric acids), and free amino acids in freeze-dried pulp were quantified by high-performance liquid chromatography (HPLC) [19]. Sugar analysis was performed using a carbohydrate analysis column with refractive index (RI) detection. Organic acids were separated on a C18 reversed-phase column and monitored by UV detection at 210 nm. For free amino acids, pre-column derivatization with phenyl isothiocyanate (PITC) was employed prior to separation, with detection at 254 nm [20]. Detailed chromatographic parameters are compiled in Table S1, and method validation characteristics—including linearity, limits of detection (LOD) and quantification (LOQ), precision, and recovery—are summarized in Table S2. All analyses were performed in triplicate, and data are reported as mg·kg^−1^ dry weight (DW).

### 2.4. Determination of Limonoids, Flavonoids, and Phenolic Acids

Using the same batch of freeze-dried and hot-air-dried Huyou tissue powders, targeted secondary metabolites—including limonoids, flavonoids, and phenolic acids—were sequentially extracted and purified [21,22]. Briefly, tissue powders were mixed with aqueous methanol solvent, homogenized, and subjected to ultrasonic extraction at ambient temperature. After centrifugation, supernatants were collected for further purification. The extraction solvents and purification protocols were individually optimized according to the physicochemical properties of each class of target compounds. Prior to instrumental analysis, all extracts were filtered through 0.2 μm polytetrafluoroethylene membranes and stored at −80 °C in amber vials to prevent photodegradation.

Limonoids were quantified using an HPLC system with a C18 column and UV detection at 210 nm. Flavonoids were profiled via UPLC-MS/MS with a C18 column under gradient elution, using electrospray ionization in positive ion mode (ESI+) and multiple reaction monitoring (MRM). Phenolic acids were determined by UPLC-UV, with detection wavelengths set at 260 nm for protocatechuic, p-hydroxybenzoic, and vanillic acids, and 320 nm for caffeic, *p*-coumaric, sinapic, and ferulic acids.

Compound identification was confirmed by comparing retention times and UV-Vis spectra against authentic standards, and quantification was performed using the external standard method. Detailed chromatographic conditions are provided in Table S3, while method validation parameters, including linearity, LOD, LOQ, precision, and recovery, are summarized in Table S4. All analyses were conducted in triplicate, and results are reported as mg·kg^−1^ dry weight (DW).

### 2.5. Assessment of Antioxidant Capacity

The identical freeze-dried and hot-air-dried Huyou tissue powders were used to evaluate antioxidant capacity via DPPH, ABTS, and FRAP assays according to commercial kit protocols (Suzhou Mengxi Biomedical Technology Co., Ltd., Suzhou, China) [23,24]. All analyses were performed in triplicate. Briefly, approximately 0.05–0.10 g of powder was homogenized in 1 mL of ice-cold 80% aqueous methanol containing 0.1% HCl. The mixture was ultrasonicated for 30 min at 4 °C, followed by centrifugation at 10,000× *g* for 10 min at 4 °C. The resulting supernatant was collected and stored at −80 °C in the dark until further analysis.

The DPPH radical scavenging activity was evaluated by monitoring the decrease in absorbance at 515 nm. ABTS radical cation scavenging capacity was measured at 734 nm. The ferric reducing antioxidant power (FRAP) was determined at 593 nm based on the reduction in the Fe^3+^-TPTZ complex to its ferrous form (Fe^2+^-TPTZ). Detailed reaction protocols and incubation conditions are summarized in Table S5.

### 2.6. Data Statistics

Statistical analyses were performed using SPSS software (version 26.0). Data processing and graphical visualization were performed in Microsoft Excel 2019 and GraphPad Prism 9.0. All experimental measurements are presented as the mean ± standard deviation (SD) derived from three independent biological replicates. Independent Student’s *t*-tests were used for inter-cultivar comparisons under identical tissue and drying conditions, while paired Student’s *t*-tests were applied to compare the two drying treatments within each biological replicate. The strength of associations between metabolite classes (flavonoids, phenolic acids, limonoids, and amino acids) and antioxidant capacity indices was determined via Pearson correlation coefficient analysis. Statistical significance was set at *p* < 0.05.

## 3. Results

### 3.1. Physicochemical Characteristics of Huyou Fruits

**Figure 2:**
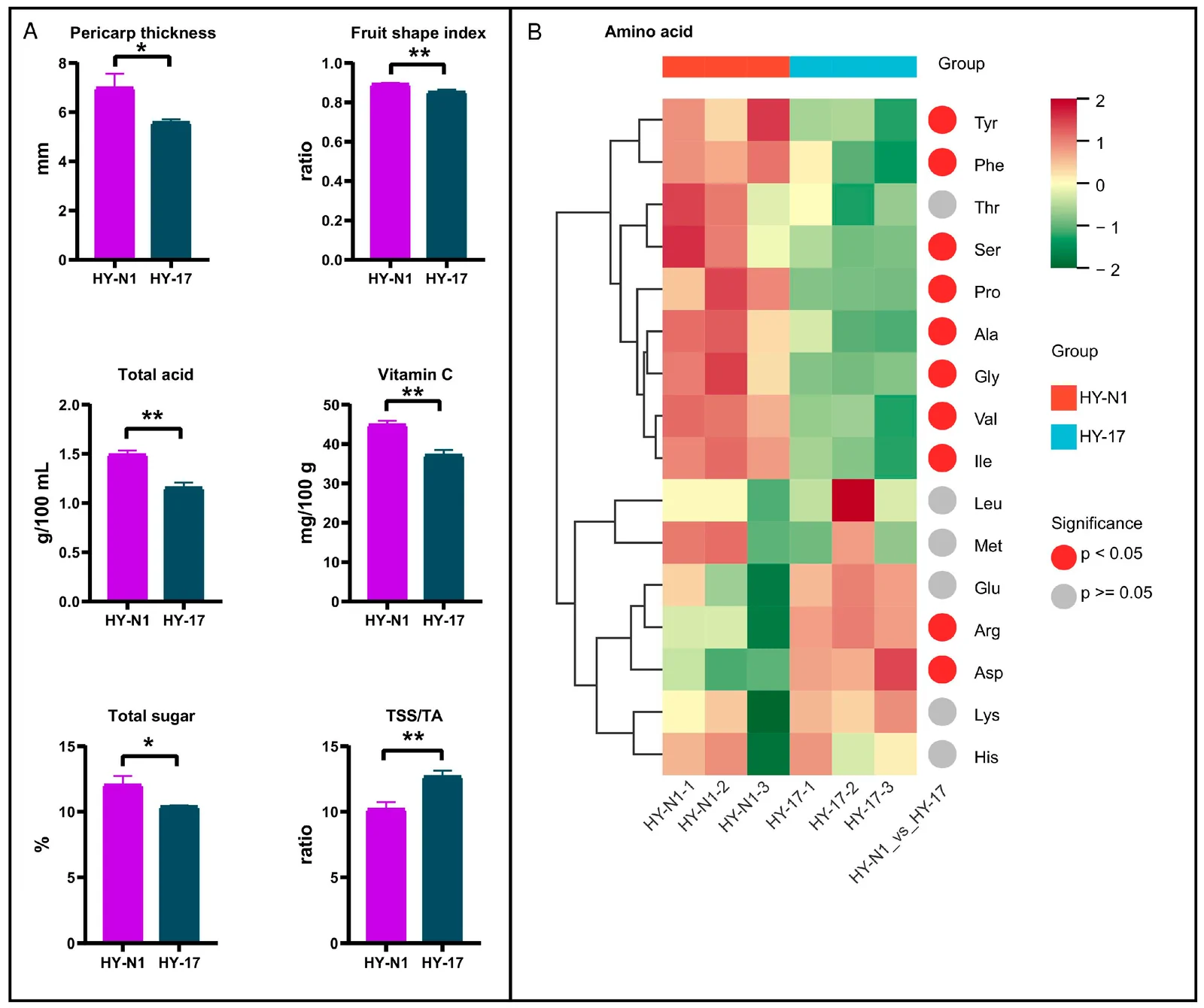
Physicochemical traits and amino acid profiles of Huyou cultivars (HY-N1 vs. HY-17). (**A**) Morphological and internal quality parameters. HY-N1 had a thicker pericarp, higher acidity, vitamin C, and sugar levels, whereas HY-17 exhibited a higher TSS/TA ratio, indicating a sweeter taste. Asterisks denote significant differences (* *p* < 0.05, ** *p* < 0.01; Student’s *t*-test). (**B**) Heatmap of free amino acids in the pulp. Color intensity indicates Z-score normalized abundance. HY-N1 was characterized by proline (Pro) accumulation, while HY-17 showed enrichment in arginine (Arg) and aspartic acid (Asp). Significant differences (*p* < 0.05) are marked.

To identify the intrinsic quality differences between the two dominant Huyou cultivars, HY-N1 and HY-17, comprehensive phenotypic and biochemical profiles were obtained (Table S6, Figure 2). Macroscopic analysis revealed no statistically significant differences (*p* > 0.05) in single fruit weight (279.85 g vs. 242.80 g), transverse/longitudinal diameters (85.10/75.86 mm vs. 82.24/70.19 mm), or edible rate (64.54% vs. 67.66%). However, marked morphological differences were observed. HY-N1 exhibited a significantly higher fruit shape index (0.89 vs. 0.85; *p* < 0.05) and thicker pericarp (6.98 vs. 5.58 mm; *p* < 0.05), whereas HY-17 demonstrated higher juice yield (41.27% vs. 35.49%; *p* < 0.05) and slightly brighter peel color (L*: 74.10 vs. 72.02).

Internal biochemical assessment indicated that both cultivars had reached commercial maturity with comparable total soluble solids (~15%). Notably, HY-N1 was characterized by significantly higher titratable acidity (1.49 vs. 1.16 g·100 mL^−1^; *p* < 0.05), vitamin C content (44.90 vs. 37.17 mg·100 g^−1^; *p* < 0.05), and total sugar levels (12.07% vs. 10.40%; *p* < 0.05) (Figure 2A). This resulted in a significantly lower SSC-to-TA ratio for HY-N1 (10.21 vs. 12.69 for HY-17; *p* < 0.05), suggesting a more tart flavor profile compared to the sweeter, more balanced HY-17.

Edible pulp chemistry further supported this divergence (Table S6). Soluble sugars were predominantly composed of sucrose, glucose, and fructose, with no significant inter-cultivar variations observed. Conversely, the higher acidity in HY-N1 was primarily attributable to a substantially larger citric acid pool (75.68 vs. 68.17 mg·g^−1^ DW; *p* < 0.05), while levels of tartaric, malic, and shikimic acids remained comparable. The free amino acid profile served as a distinctive metabolic fingerprint (Figure 2B). HY-N1 accumulated significantly higher total amino acids (22891.01 vs. 20358.59 mg·kg^−1^ DW; *p* < 0.05), largely due to a substantially higher proline content (7617.25 vs. 3370.99 mg·kg^−1^ DW; ~2.3-fold). In contrast, HY-17 exhibited significantly higher levels of aspartic acid (5372.43 vs. 4460.50 mg·kg^−1^ DW) and arginine (7605.60 vs. 6613.14 mg·kg^−1^ DW). These findings indicate a clear metabolic pattern: HY-N1 showed proline accumulation along with higher acidity and nutrient density, whereas HY-17 favored arginine and aspartic acid, consistent with its sugar-acid ratio.

### 3.2. Tissue-Specific Secondary Metabolite Architecture

#### 3.2.1. Tissue-Specific Accumulation of Limonoids Reveals Enhanced Biosynthesis in HY-N1

**Figure 3:**
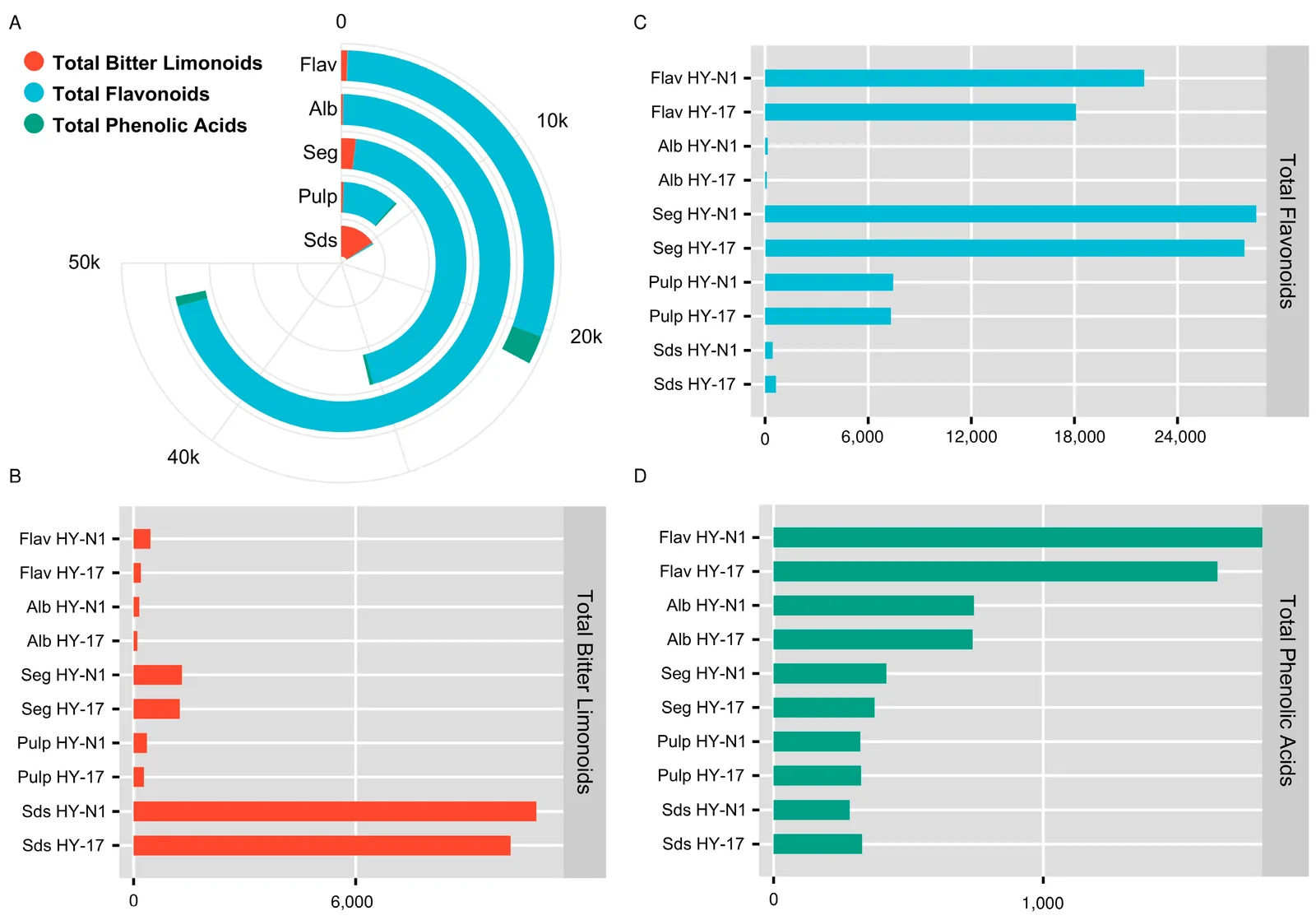
Tissue-specific secondary metabolite architecture of two Huyou cultivars. (**A**) Limonoid distribution. Seeds were the primary reservoir; HY-N1 accumulated significantly higher levels in flavedo and pulp than HY-17 (*p* < 0.05). (**B**) Heatmap of total limonoids (Z-score normalized). Levels peaked in seeds and segment membranes, largely sequestered from edible tissues, with HY-N1 showing consistently elevated accumulation across peripheral and edible fractions. (**C**) Total flavonoid allocation. HY-N1 prioritized bitter flavanone glycosides (naringin, neohesperidin) in albedo and segment membranes, whereas HY-17 favored seed translocation and polymethoxyflavone (tangeretin, nobiletin) enrichment in peripheral layers. (**D**) Total phenolic acid distribution. Ferulic acid dominated (>60% of total in flavedo); HY-N1 concentrated acids in flavedo, while HY-17 showed superior translocation to seeds and pulp (*p* < 0.05).

The distribution of the primary bitter limonoids, limonin and nomilin, exhibited a highly conserved, tissue-specific pattern in both cultivars (Table S7 and Figure 3A,B). Both compounds were predominantly concentrated in the non-edible tissues. Seeds served as the primary metabolic sink, accumulating remarkably high limonin concentrations (8437.65–8751.99 mg·kg^−1^ DW) without significant inter-cultivar variation (Figure S1). However, HY-N1 had significantly higher nomilin concentrations in seeds (2444.77 vs. 1434.40 mg·kg^−1^ DW in HY-17; *p* = 0.003). This cultivar difference was also evident in the non-edible peripheral tissues and edible pulp. HY-N1 consistently accumulated higher limonoid levels across these tissues. Notably, limonin in HY-N1 pulp (339.44 mg·kg^−1^ DW) was significantly higher than in HY-17 (267.93 mg·kg^−1^ DW; *p* = 0.022), a disparity that was even more pronounced in the flavedo (339.99 vs. 72.60 mg·kg^−1^ DW; *p* = 0.049). Similarly, nomilin levels in HY-N1 were significantly higher in both pulp (20.67 vs. 11.33 mg·kg^−1^ DW; *p* = 0.002) and seeds. Collectively, these results indicate a distinct metabolic profile in HY-N1: consistently higher levels of bitter limonoid accumulation, particularly in the fruit periphery and seeds.

#### 3.2.2. Divergent Flavonoid Allocation Patterns Define Cultivar-Specific Metabolic Profiles

**Figure 4:**
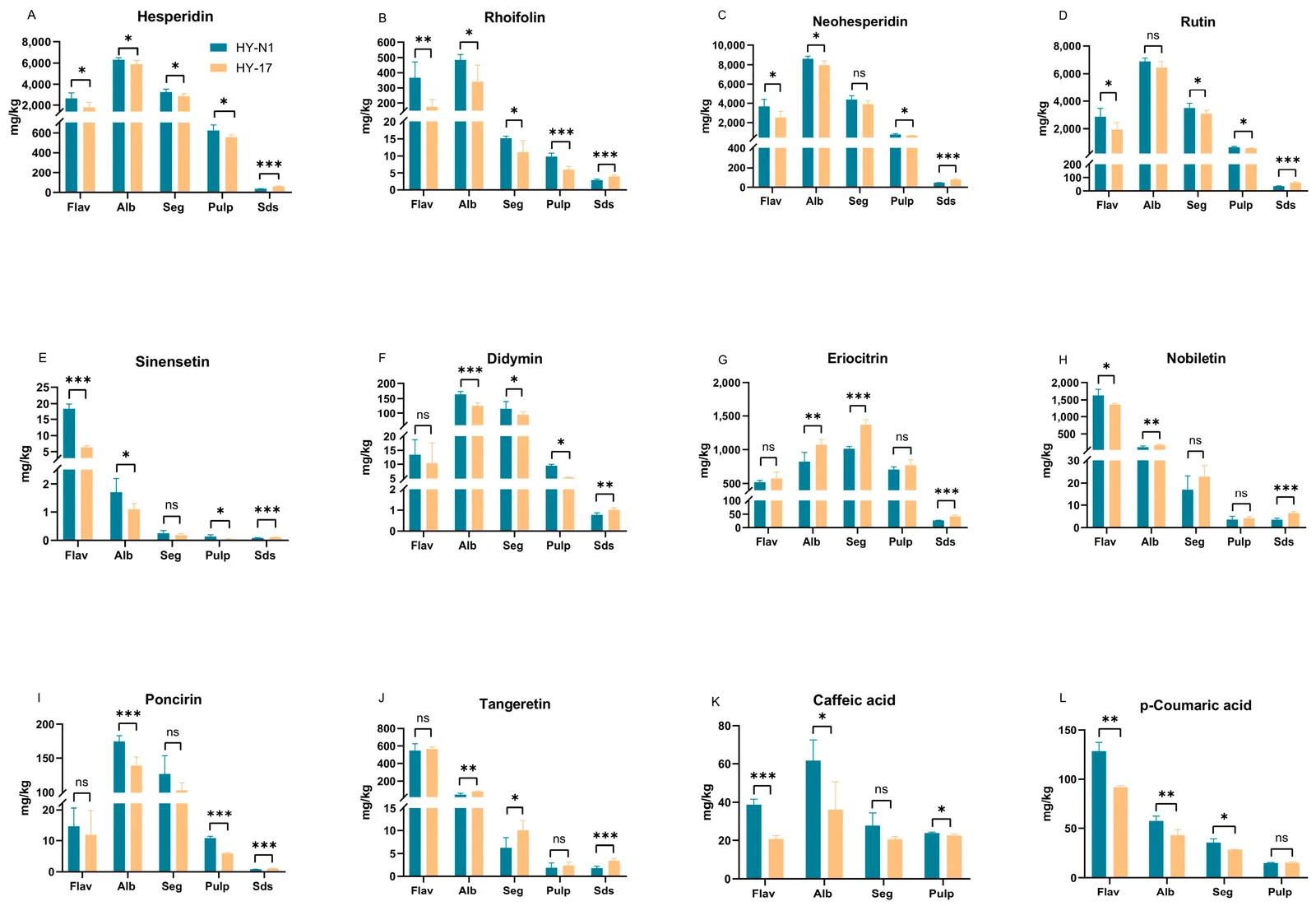
Tissue-specific distribution of individual flavonoids and phenolic acids in Huyou fruits (HY-N1 vs. HY-17). Bar charts showing concentration (mg·kg^−1^ DW) of 12 individual compounds across five fruit tissues (flavedo, albedo, segment membranes, pulp, and seeds) of HY-N1 and HY-17 cultivars. (**A**) Hesperidin, (**B**) Rhoifolin, (**C**) Neohesperidin, (**D**) Rutin, (**E**) Sinensetin, (**F**) Didymin, (**G**) Eriocitrin, (**H**) Nobiletin, (**I**) Poncirin, (**J**) Tangeretin, (**K**) Caffeic acid, (**L**) p-Coumaric acid. Data are presented as mean ± SD (n = 3). Asterisks indicate significant differences between cultivars (* *p* < 0.05, ** *p* < 0.01, *** *p* < 0.001; ns, not significant).

Flavonoids exhibited a pronounced spatial gradient, with the highest accumulation in the albedo and segment membranes, followed by the flavedo, while the pulp and seeds showed lower levels (Table S7, Figure 3C). Water-soluble flavanone glycosides (e.g., naringin, neohesperidin, narirutin) accounted for over 80% of the total flavonoid pool in the peel and membranes (Figure 4), whereas lipophilic polymethoxyflavones (e.g., tangeretin, nobiletin) were minor constituents. Notably, several aglycones (e.g., hesperetin, naringenin) remained below detection limits, confirming glycosylation as the primary storage form. While HY-N1 accumulated significantly higher total flavonoids in the flavedo and albedo (*p* < 0.001), no significant inter-cultivar differences were observed in the segment membranes or pulp. Conversely, HY-17 exhibited a different pattern of seed enrichment, accumulating 43% more total flavonoids in the seeds than HY-N1 (*p* < 0.001).

Clear differences were observed between the two cultivars in the tissue accumulation patterns of specific flavonoids. HY-N1 accumulated significantly higher levels of flavanone glycosides in the albedo and segment membranes. Specifically, HY-N1 accumulated significantly higher levels of key flavanone glycosides in the albedo, including naringin (albedo: 13,580.56 vs. 12,686.32 mg·kg^−1^ DW, *p* = 0.032) and neohesperidin (albedo: 8610.68 vs. 7961.02 mg·kg^−1^ DW, *p* = 0.012). This pattern was also observed for polymethoxyflavones, with HY-N1 exhibiting higher accumulation of sinensetin (flavedo: 18.39 vs. 6.37 mg·kg^−1^ DW; *p* < 0.001) and nobiletin (flavedo: 1629.92 vs. 1364.55 mg·kg^−1^ DW; *p* = 0.013) (Figure 4).

In contrast, HY-17 exhibited a different accumulation pattern with higher levels of specific flavonoids in the seeds and peripheral peel tissues. HY-17 accumulated significantly higher levels of specific polymethoxyflavones and minor glycosides in the albedo, including tangeretin (81.17 vs. 45.56 mg·kg^−1^ DW; *p* = 0.002) and nobiletin (175.47 vs. 114.14 mg·kg^−1^ DW; *p* = 0.005). This trend was also evident for eriocitrin, which was significantly more abundant in both the albedo (1072.23 vs. 820.77 mg·kg^−1^ DW; *p* = 0.005) and seeds (42.16 vs. 26.58 mg·kg^−1^ DW; *p* < 0.001) of HY-17 (Figure 4). These findings show that, while HY-N1 had higher levels of bulk bitter glycosides in the albedo, HY-17 exhibited higher levels of flavonoids in the seeds.

#### 3.2.3. Divergent Phenolic Acid Allocation Mirrors Flavonoid Patterns

Phenolic acids displayed a clear spatial gradient, with the highest accumulation in the flavedo declining progressively towards the inner fruit tissues (Table S7, Figure 3D). Ferulic acid was the predominant constituent in both cultivars, accounting for over 60% of the total phenolic acid pool in the flavedo (HY-N1: 1208.93 vs. HY-17: 976.59 mg·kg^−1^ DW). Other major forms included vanillic acid, p-hydroxybenzoic acid, and p-coumaric acid, whereas protocatechuic acid and caffeic acid were present at relatively low levels. Consistent with the distribution patterns of flavonoids and limonoids, the edible pulp contained the lowest phenolic acid levels (Figure 4K,L).

Clear differences between the two cultivars were evident in this accumulation pattern. HY-N1 showed higher concentrations of phenolic acids in the peel tissues. It had significantly higher total phenolic acid content in the flavedo (1970.58 vs. 1646.24 mg·kg^−1^ DW; *p* < 0.001), largely due to substantially higher ferulic acid (1208.93 vs. 976.59 mg·kg^−1^ DW; *p* < 0.001). This pattern was also observed for vanillic acid (167.25 vs. 153.45 mg·kg^−1^ DW; *p* = 0.009) and p-coumaric acid (128.33 vs. 91.93 mg·kg^−1^ DW; *p* = 0.003). Furthermore, HY-N1 maintained higher concentrations of caffeic acid in both the flavedo (38.74 vs. 20.78 mg·kg^−1^ DW; *p* < 0.001) and albedo (61.69 vs. 36.29 mg·kg^−1^ DW; *p* = 0.033).

In contrast, HY-17 showed higher phenolic acid accumulation in the seeds and edible pulp. Although total phenolic acids in the flavedo were lower, HY-17 achieved significantly higher accumulation in these tissues: specifically, higher ferulic acid in the pulp (182.94 vs. 173.20 mg·kg^−1^ DW; *p* = 0.014) and p-coumaric acid in the seeds (105.61 vs. 94.53 mg·kg^−1^ DW; *p* = 0.004). Notably, the total phenolic acid content in the seeds was markedly higher in HY-17 (328.14 vs. 281.97 mg·kg^−1^ DW; *p* < 0.001). This convergent pattern parallels the divergent flavonoid accumulation patterns, indicating a consistent metabolic difference between the two cultivars.

### 3.3. Tissue-Specific Antioxidant Capacity Reflects Cultivar-Specific Metabolite Profiles

#### 3.3.1. Tissue-Specific Antioxidant Capacity Differs Between Cultivars

Antioxidant capacity varied substantially across fruit tissues and differed between cultivars (Table S8). The FRAP assay showed a clear spatial gradient, with the flavedo and albedo having the highest capacities among non-edible tissues (9.75–29.73 μmol TE·g^−1^ DW), followed by the pulp (7.62–19.79 μmol TE·g^−1^ DW), and seeds showing the lowest levels (4.00–4.15 μmol TE·g^−1^ DW). In contrast, the DPPH assay indicated that the albedo and segment membranes had the highest values, reaching up to 74.92 μmol TE·g^−1^ DW. The ABTS assay, however, showed a more uniform distribution, suggesting it reflected a broader spectrum of hydrophilic and lipophilic antioxidants than FRAP and DPPH.

Clear differences between cultivars were observed in this antioxidant profile. HY-N1 consistently had significantly higher FRAP values in the edible pulp (19.79 vs. 7.62 μmol TE·g^−1^ DW; *p* = 0.006), flavedo (20.67 vs. 13.60 μmol TE·g^−1^ DW; *p* = 0.017), and segment membranes (15.13 vs. 9.75 μmol TE·g^−1^ DW; *p* = 0.006). Conversely, HY-17 tended to have higher, though not significant, FRAP values in the albedo (29.73 vs. 16.12 μmol TE·g^−1^ DW; *p* > 0.05) and higher DPPH scavenging in the albedo (74.92 vs. 52.73 μmol TE·g^−1^ DW) and segment membranes (47.45 vs. 32.18 μmol TE·g^−1^ DW). Notably, HY-N1 had higher DPPH scavenging in the seeds (7.29 vs. 5.66 μmol TE·g^−1^ DW; *p* < 0.001). In contrast, the ABTS assay showed no significant differences between cultivars across any tissue, indicating that the total peroxyl radical scavenging capacity is largely similar between cultivars. Together, these results show that the antioxidant advantage of HY-N1 is both assay- and tissue-dependent, mainly appearing as higher reducing power (FRAP) and higher seed radical scavenging (DPPH), rather than a general increase across all tissues.

#### 3.3.2. Antioxidant Capacity Is Associated with the Differential Allocation of Specific Metabolite Classes

Correlation analysis revealed the metabolic basis of the observed antioxidant profiles, showing a clear contrast in the contributions of distinct phytochemical families (Figure S2). Bitter limonoids (limonin and nomilin) exhibited significant negative correlations with all three antioxidant assays (|r| = 0.56–0.78, *p* < 0.001). This inverse relationship indicates that tissues with high limonoid accumulation, such as the seeds, had lower measured radical scavenging capacities under these assay conditions.

In contrast, flavonoids and phenolic acids were the primary contributors to antioxidant capacity. Among the flavanones, neoeriocitrin, rutin, hesperidin, and naringin showed the strongest positive correlations (r = 0.77–0.86, *p* < 0.0001). Notably, total flavonoid content was a robust predictor of antioxidant potential, particularly for ABTS scavenging (r = 0.990, *p* < 0.0001). Similarly, total phenolic acids correlated highly with DPPH and ABTS activities (r > 0.89, *p* < 0.0001), with p-hydroxybenzoic acid, vanillic acid, and caffeic acid being key individual contributors. Conversely, polymethoxyflavones (tangeretin, sinensetin, nobiletin) showed weaker or non-significant correlations, indicating a smaller direct role in the measured antioxidant mechanisms compared with their glycosylated counterparts, despite their recognized bioactivities.

### 3.4. Differential Thermal Stability of Phytochemicals Leads to Functional Changes

#### 3.4.1. Differential Thermal Stability of Amino Acids

**Figure 5:**
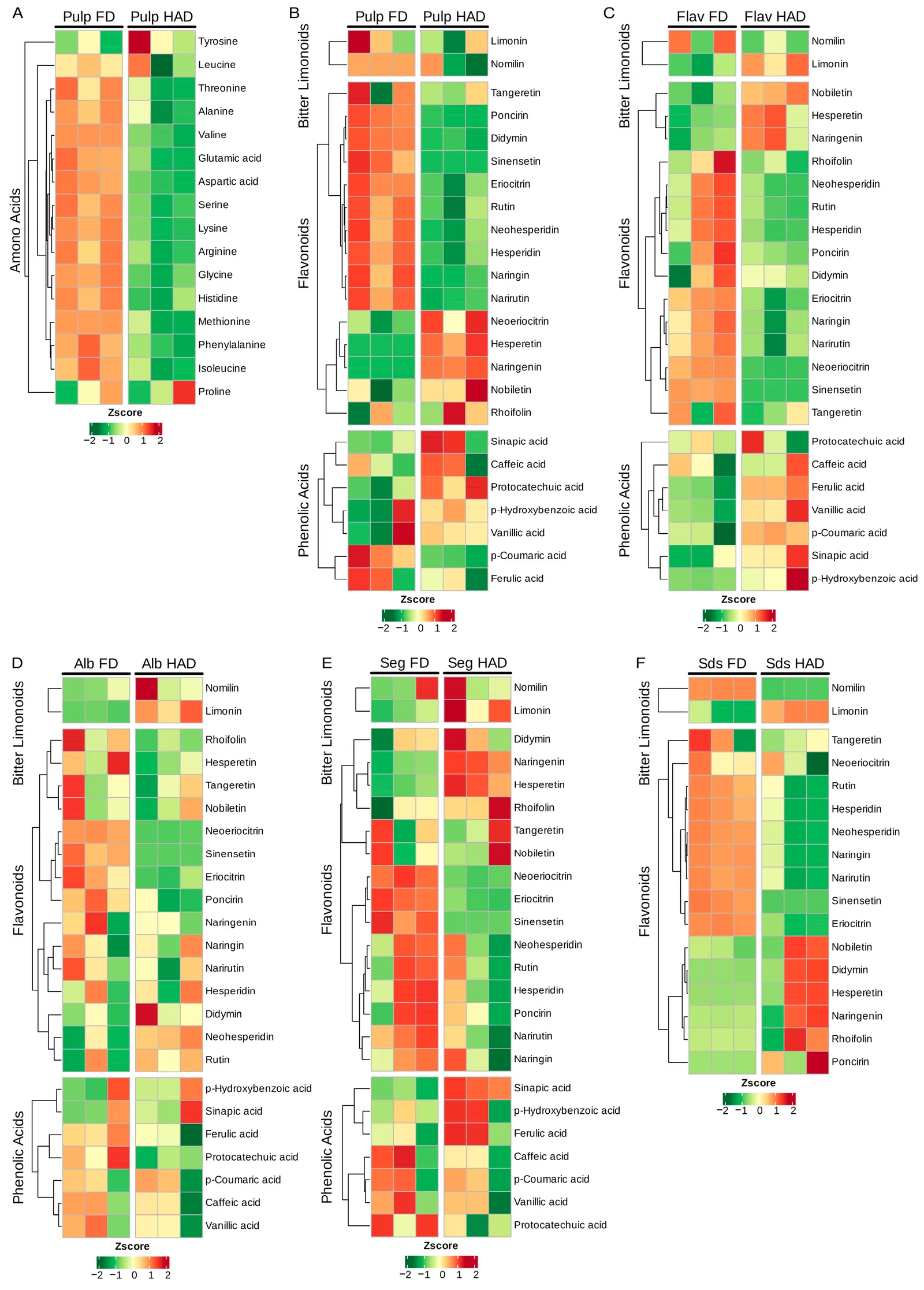
Effect of hot-air drying on the metabolic profile of Huyou fruits (HY-N1 and HY-17). Heatmaps showing fold-change (Z-score normalized) in metabolite abundance between freeze-dried (FD) and hot-air dried (HAD) samples across multiple fruit tissues. (**A**) Changes in total free amino acid profile in the edible pulp after hot-air drying. (**B**–**F**) Comparative heatmaps of secondary metabolite abundance (bitter limonoids, flavonoids, and phenolic acids) between FD and HAD treatments in (**B**) pulp, (**C**) flavedo, (**D**) albedo, (**E**) segment membranes, and (**F**) seeds.

Hot-air drying significantly reduced the total free amino acid pool, resulting in lower protein nutritional value of Huyou fruits. HY-N1 lost 21.6% of its total content (from 22,809.89 to 17,883.69 mg·kg^−1^ DW), while HY-17 lost 22.5% (from 20,468.97 to 15,866.84 mg·kg^−1^ DW) (Table S9, Figure 5). This depletion resulted mainly from the thermal degradation of acidic and sulfur-containing amino acids. Aspartic acid and lysine showed the greatest reductions; for instance, aspartic acid in HY-N1 dropped by 47.4% (from 4460.50 to 2344.28 mg·kg^−1^ DW), and lysine decreased by 61.6% (HY-N1) and 52.1% (HY-17). Methionine was also highly heat-labile, with retention rates of only 33.7% (HY-N1) and 64.7% (HY-17).

In contrast, proline showed remarkable thermal stability, with its concentration remaining nearly unchanged or slightly higher (102.5% in HY-N1; 105.5% in HY-17). This observation, coupled with the increase in hydrophobic amino acids like isoleucine and leucine in HY-17 (121.7% and 140.0% retention, respectively), may be associated with early-stage Maillard reactions. The condensation of reducing sugars with reactive amino groups (e.g., lysine) would make those amino acids undetectable by the analytical method, thereby increasing the relative proportions of stable amino acids like proline and hydrophobic branched-chain amino acids. Furthermore, threonine and valine were better retained in HY-17 than in HY-N1, suggesting that genetic background influences amino acid stability during thermal processing.

#### 3.4.2. Flavonoid Stability During Thermal Processing Shows Structural Dependence

Hot-air drying induced significant, structure-dependent alterations in the flavonoid profile, particularly in the pulp, flavedo, and seeds (Figure 5). The albedo maintained relatively stable total flavonoid levels, with only slight decreases of 6.6% in HY-N1 and 19.4% in HY-17. The seeds showed the most severe loss, where total flavonoids in HY-N1 declined by 40.9% and in HY-17 by 70.4%. Neohesperidin in the pulp decreased by 29.2% in HY-N1 (from 803.58 to 568.80 mg·kg^−1^ DW) and 26.0% in HY-17 (from 689.88 to 510.56 mg·kg^−1^ DW). Naringin in the seeds was severely degraded, declining by 54.3% in HY-N1 and 75.8% in HY-17. Similarly, rutin in the seeds decreased by 36.8% in HY-N1 and 77.2% in HY-17, while eriocitrin in the pulp of HY-17 was reduced by 37.0%. Conversely, lipophilic polymethoxyflavones apparently increased in the flavedo. Nobiletin in the flavedo of HY-N1 rose by 20.9% (from 1629.92 to 1970.86 mg·kg^−1^ DW) and in HY-17 by 14.7% (from 1364.55 to 1565.16 mg·kg^−1^ DW). However, sinensetin was completely degraded and not detected in any tissue after hot-air drying. Together, these results highlight a clear structure-localization relationship: glycosylated flavonoids degrade readily in inner tissues, whereas polymethoxyflavones in the protective flavedo are preserved or concentrated.

#### 3.4.3. Phenolic Acids Increase, Presumably Linked to Matrix Effects

Contrary to the generalized degradation of amino acids and flavanone glycosides, total phenolic acids increased significantly across all tissues after hot-air drying (Table S9, Figure 5). In the flavedo, total phenolic acids in HY-N1 rose from 1970.58 to 4486.38 mg·kg^−1^ DW, while in HY-17 it increased from 1646.24 to 3481.80 mg·kg^−1^ DW. Substantial increases were also observed in the albedo, with HY-N1 rising from 742.81 to 1437.25 mg·kg^−1^ DW and HY-17 from 737.75 to 1413.39 mg·kg^−1^ DW. The segment membranes also showed marked increases, with HY-N1 increasing from 418.24 to 871.31 mg·kg^−1^ DW and HY-17 from 374.17 to 812.80 mg·kg^−1^ DW. Even the edible pulp showed a near-doubling of total phenolic acids in both cultivars (HY-N1 from 321.44 to 646.77 mg·kg^−1^ DW; HY-17 from 324.15 to 639.91 mg·kg^−1^ DW).

This overall increase was largely associated with the behavior of major acids. Ferulic acid, the dominant constituent, contributed to this increase, rising by 14.7% in the HY-N1 flavedo. However, the stability of individual phenolic acids varied. Protocatechuic acid showed relative instability in specific tissues; for instance, it decreased by 21.5% in the flavedo of HY-17 and by 12.8% in the albedo of HY-N1. Caffeic acid also exhibited tissue-specific sensitivity, notably decreasing in the flavedo of HY-17 from 20.78 to 16.68 mg·kg^−1^ DW.

#### 3.4.4. Antioxidant Capacity Changes Depending on Assay Chemistry

Hot-air drying led to changes in Huyou’s antioxidant capacity, with outcomes depending on assay chemistry, tissue type, and cultivar-specific metabolite profiles (Table S10, Figure S3). In the FRAP assay, the pulp of HY-N1 decreased significantly from 19.79 to 8.37 μmol TE·g^−1^ DW (*p* < 0.01), whereas HY-17 remained stable (7.62 vs. 8.23 μmol TE·g^−1^ DW). Conversely, the flavedo of both cultivars increased significantly, with HY-N1 rising from 20.67 to 31.98 μmol TE·g^−1^ DW and HY-17 from 13.60 to 35.07 μmol TE·g^−1^ DW (*p* < 0.05). Segment membranes showed opposite trends: HY-N1 decreased (15.13 to 10.79 μmol TE·g^−1^ DW) while HY-17 increased (9.75 to 16.85 μmol TE·g^−1^ DW) (*p* < 0.05).

Results from the DPPH assay largely followed this pattern. The flavedo of both cultivars showed marked increases after hot-air drying, with HY-N1 rising from 56.27 to 79.14 μmol TE·g^−1^ DW and HY-17 from 53.98 to 100.51 μmol TE·g^−1^ DW. Segment membranes of HY-17 decreased significantly (47.45 to 33.52 μmol TE·g^−1^ DW), while the pulp and albedo showed no consistent significant changes across cultivars. The ABTS assay was largely unaffected by hot-air drying. Most tissues exhibited only minor fluctuations. The pulp of both cultivars showed a slight but significant increase (HY-N1: 39.14 to 49.60 μmol TE·g^−1^ DW; HY-17: 36.72 to 47.70 μmol TE·g^−1^ DW), while other tissues generally maintained their pre-drying levels. Consistent with these antioxidant results, the spectrophotometric measurements of total phenolic acids and total flavonoids also varied. Total phenolic acids in the flavedo increased significantly for both cultivars (HY-N1: 20.05 to 22.71 mg GAE·g^−1^ DW; HY-17: 18.55 to 19.15 mg GAE·g^−1^ DW). Total flavonoids, however, remained largely stable across all tissues, with no consistent significant degradation or increase observed.

## 4. Discussion

As a unique pomelo-dominant cultivar endemic to China, Huyou represents a valuable resource for both fresh fruit consumption and functional food development [4,8]. To address the knowledge gap regarding tissue-specific metabolic differences and processing-induced quality changes, this study used a high-resolution, tissue-resolved metabolomic strategy to systematically compare two elite cultivars (HY-N1 and HY-17) and evaluate their differential responses to hot-air drying. Our results revealed clear metabolic differences between the two cultivars and demonstrated the impact of drying conditions on the retention of key bioactive compounds and antioxidant capacity. These findings provide insights for cultivar selection and by-product utilization, enabling the exploration of cultivar-specific end-use orientations based on their intrinsic phytochemical profiles, and establishing a scientific basis for quality evaluation, processing optimization, and high-value use of Huyou by-products [25,26].

Physicochemical characterization of HY-N1 and HY-17 confirmed a clear difference in quality traits. While basic morphological traits were similar, HY-N1 exhibited a thicker pericarp and higher accumulation of primary metabolites, including sugars, organic acids, and vitamin C, pointing to a nutrient-dense metabolic phenotype [9,16]. In contrast, HY-17 showed consumer-oriented traits, such as higher juice yield, brighter color, and a balanced TSS/TA ratio [18]. Amino acid profiling further supported this difference: HY-N1’s proline dominance may reflect enhanced abiotic stress tolerance, whereas HY-17’s enrichment in arginine and aspartic acid suggests distinct nitrogen accumulation patterns favoring seed development [4]. These metabolic differences point to distinct prospective end-use orientations for the two cultivars and offer useful references for cultivar utilization.

Based on the primary metabolic differences between HY-N1 and HY-17, our tissue-resolved secondary metabolite profiling revealed a tissue-specific compartmentalization of bioactive compounds [25]. These compounds were preferentially accumulated in peripheral non-edible tissues—flavedo, albedo, and segment membranes—where phenylpropanoid biosynthesis contributes to chemical defense against biotic stress, while bitter limonoids were mostly sequestered away from the edible pulp [26]. HY-N1 showed higher limonin in the pulp, which may contribute to pest resistance, whereas HY-17 had bitterness mostly restricted to seeds and peripheral tissues, consistent with fresh-consumption quality [27]. Correspondingly, HY-N1 accumulated high levels of bitter flavanone glycosides in processing by-products (albedo, segment membranes), while HY-17 showed higher arginine-associated flavonoids and phenolic acids in seeds and edible pulp [28]. These findings not only refine previous compositional surveys of Huyou but also provide a framework for the structure-dependent thermal stability and processing-induced changes detailed in subsequent sections.

The spatial distribution of antioxidant capacity in Huyou directly mirrored the tissue-specific accumulation of secondary metabolites, consistent with our third hypothesis that antioxidant changes depend on both tissue type and the chemical mechanism of the assay [6,18]. As anticipated, the flavedo, albedo, and segment membranes—tissues enriched in flavonoids and phenolic acids—exhibited the highest antioxidant activities, whereas the seeds and pulp showed minimal capacity (Figure S3, Table S8). Importantly, HY-N1 demonstrated significantly higher antioxidant capacity across these peripheral and edible tissues compared to HY-17, consistent with its greater accumulation of total phenolics [29]. Correlation analysis identified the chemical basis of this difference: flavonoids and phenolic acids were the primary positive contributors, showing strong correlations with ABTS scavenging (r > 0.89, *p* < 0.0001), whereas bitter limonoids exhibited significant negative correlations with all assays (|r| = 0.56–0.78, *p* < 0.01) [18]. This inverse relationship suggests that higher levels of bitter limonoid compounds are associated with lower radical scavenging capacity under these assay conditions [6]. Consequently, the flavedo and albedo represent optimal targets for functional ingredient extraction due to their superior phytochemical density, while the segment membranes constitute a promising yet underutilized by-product source [30]. Although seeds possess lower antioxidant capacity, their uniquely high limonoid content offers potential for use in high-value bitter nutraceuticals. By establishing this structure–activity framework, we provide a basis for interpreting the differential thermal stability of antioxidant capacity described in the following section [7].

Extending this framework, hot-air drying induced significant changes in Huyou tissues, consistent with our second hypothesis that thermal stability may depend on molecular structure and matrix-related properties [29]. Unlike the gentle preservation offered by freeze-drying, thermal processing led to a cascade of chemical transformations. Amino acids exhibited a striking structure-dependent pattern: while heat-labile aspartic acid, lysine, and methionine underwent severe degradation (>50% loss) likely via Maillard reactions or oxidative decarboxylation, proline remained remarkably stable due to its cyclic conformation, which may confer resistance to thermal denaturation. This differential stability parallels the cultivar-specific accumulation patterns observed previously [31]. Flavonoids further exemplified this principle: hydrophilic flavanone glycosides (e.g., neohesperidin, naringin) were vulnerable to thermal hydrolysis, particularly in the hydrated pulp and seeds, whereas lipophilic polymethoxyflavones (e.g., nobiletin) appeared to increase in the flavedo [32]. This apparent increase in PMFs likely reflects the thermal release of cell wall-bound flavonoids rather than de novo synthesis, a phenomenon related to the lipophilic matrix of the peel [33]. Furthermore, the albedo acted as a thermal buffer, maintaining stable total flavonoid levels due to its dense parenchymatous structure. Phenolic acids displayed the most interesting response: total contents surged in the flavedo and albedo, likely due to the heat-accelerated hydrolysis of ester-linked, insoluble bound phenolics into free, extractable forms—effectively releasing the structural antioxidants of the cell wall. Consequently, antioxidant capacity changes followed a strictly assay-dependent pattern [34]. The FRAP and DPPH assays, reliant on single-electron transfer mechanisms, were highly sensitive to these transformations, showing increases in the flavedo (related to PMF and phenolic acid release) but decreases in the HY-N1 pulp (related to glycoside degradation). In contrast, the ABTS assay, measuring broad-spectrum peroxyl radical scavenging via hydrogen atom transfer, remained largely stable, whereas FRAP and DPPH outputs exhibited tissue- and cultivar-dependent increases or decreases [35]. Together, these findings demonstrate that hot-air drying does not uniformly degrade quality but rather selectively alters the phytochemical profile in a predictable, structure-dependent manner, providing a basis for optimizing drying protocols to either preserve labile nutrients or enhance the extractability of structural bioactives [36].

Future work should pursue several complementary research directions to support the full-scale utilisation of Huyou resources. To better define cultivar end-use suitability, further studies will incorporate sensory evaluation, consumer acceptance surveys and processed-product quality assessment. Expanded cultivar panels and genetic dissection of key metabolic traits will help translate observed phytochemical variation into practical cultivar-screening targets. Multi-temperature hot-air-drying trials are also needed to establish robust processing parameters applicable for real-world industrial production. Collectively, these efforts will provide more comprehensive technical support for Huyou breeding, processing optimisation and high-value by-product valorisation.

## 5. Conclusions

This study systematically characterized the nutritional and functional profiles across five distinct tissues of two Huyou cultivars (HY-N1 and HY-17) and evaluated the impact of freeze-drying versus hot-air drying. The results reveal that HY-N1 accumulates higher levels of bioactive compounds, while HY-17 exhibits milder acidity and reduced limonoid-related bitterness according to physicochemical and phytochemical profiling. Derived from the present dataset, these metabolic traits provide valuable references for exploring their respective end-use potentials. A clear spatial gradient of metabolites was observed: flavonoids and phenolic acids were concentrated in the peel, limonoids in the seeds and membranes, and soluble sugars in the pulp. Thermal processing led to differences in stability among compound classes. Amino acids with reactive side chains (aspartic acid, lysine, methionine) were more susceptible to heat, whereas proline and lipophilic polymethoxyflavones remained stable or increased in the flavedo. Notably, hot-air drying did not trigger a universal reduction in antioxidant capacity across all samples. Antioxidant responses varied markedly depending on fruit tissue, cultivar, and analytical assay, likely driven by the compensatory release of bound phenolic acids in certain tissues alongside compound degradation in others. Overall, this work unravels the phytochemical underpinnings of Huyou quality and characterizes cultivar-specific, tissue-resolved metabolite distribution. It highlights peel and segment-membrane fractions as promising sources of functional ingredients and underscores the need for tissue- and metabolite-specific drying strategies. These findings deliver a metabolite-informed framework for cultivar screening, processing tuning, and high-value by-product valorization of this unique citrus resource.

## Data Availability

The original contributions presented in this study are included in the article/Supplementary Material. Further inquiries can be directed to the corresponding author.

## Supplementary Materials

The following supporting information can be downloaded at: https://www.mdpi.com/article/10.3390/foods15213805/s1, Figure S1: Tissue-specific distribution of limonoids, flavanone glycosides, and phenolic acids in Huyou fruits (HY-N1 vs. HY-17). Bar charts illustrate the concentrations (mg·kg^−1^ DW) of 12 additional individual compounds across five fruit tissues. (A) Limonin, (B) Nomilin, (C) Naringin, (D) Naringenin, (E) Narirutin, (F) Neoeriocitrin, (G) Hesperetin, (H) Ferulic acid, (I) Protocatechuic acid, (J) Sinapic acid, (K) Vanillic acid, (L) p-Hydroxybenzoic acid. Data are presented as mean ± SD. Asterisks indicate significant differences between cultivars (* *p* < 0.05, ** *p* < 0.01, *** *p* < 0.001; ns, not significant; ND, not detected); Figure S2: Correlation analysis between metabolite abundance and antioxidant capacity. Heatmap of Pearson correlation coefficients (*r*) between individual metabolites and antioxidant assays (FRAP, DPPH, ABTS). Bitter limonoids (limonin, nomilin) exhibited significant negative correlations (|*r*| = 0.56–0.78, *p* < 0.001), whereas flavanone glycosides (neoeriocitrin, rutin, hesperidin, naringin) and phenolic acids (p-hydroxybenzoic acid, vanillic acid, caffeic acid) showed strong positive correlations (r = 0.77–0.86, *p* < 0.0001). Total flavonoid content was the most robust predictor of ABTS scavenging (r = 0.990). Color intensity reflects the magnitude and direction of correlation. Asterisks denote significance levels; Figure S3: Effect of hot-air drying on antioxidant capacity across fruit tissues of HY-N1 and HY-17. Radar charts comparing antioxidant activity (FRAP, DPPH, ABTS; µmol TE·g^−1^ DW) across five fruit tissues (albedo, flavedo, pulp, seeds, segment membranes) between freeze-dried (FD) and hot-air dried (HAD) samples. (A) FD HY-N1, (B) HAD HY-N1, (C) FD HY-17, (D) HAD HY-17; Table S1: Chromatographic conditions for analysis of soluble sugars, organic acids, and free amino acids; Table S2: Method validation parameters for analysis of soluble sugars, organic acids, and free amino acids: Table S3: Extraction procedures and instrumental parameters for analysis of limonoids, flavonoids, and phenolic acids; Table S4: Method validation parameters for analysis of limonoids, flavonoids, and phenolic acids; Table S5: Antioxidant assay protocols for DPPH, ABTS, and FRAP methods; Table S6: Comparative chemical profile of “Changhu No. 1” and “01-7 Huyou” (mean ± SD, n = 3); Table S7: Tissue-specific distribution of secondary metabolites in Huyou cultivars (mg·kg^−1^ DW); Table S8: Tissue-specific distribution of total flavonoids, total phenolic acids, and antioxidant capacity in Huyou cultivars (mean ± SD); Table S9: Effect of drying methods on secondary metabolites in Huyou cultivars (mean ± SD); Table S10: Effect of drying methods on total flavonoids, total phenolic acids, FRAP, DPPH and ABTS in Huyou fruits (mean ± SD).

## Abbreviations

The following abbreviations are used in this manuscript:

ABTS	2,2′-azino-bis(3-ethylbenzothiazoline-6-sulfonic acid)
DPPH	2,2-diphenyl-1-picrylhydrazyl
ESI	electrospray ionization
FRAP	ferric reducing antioxidant power
HAD	hot-air drying
HPLC	high-performance liquid chromatography
LOD	limit of detection
LOQ	limit of quantification
TSS	total soluble solids
