# Supplementary material for: Tissue-Specific Phytochemical Profiles of Two Huyou Cultivars: Bioactive Compounds and Thermal Processing Effects
Source: Foods. 2026 Oct 23;15(21):3805. doi: 10.3390/foods15213805 (PMC13762711; doi:10.3390/foods15213805)
Supplement: Supplementary file 1 [file foods-15-03805-s001.zip › Figure S2.pdf]

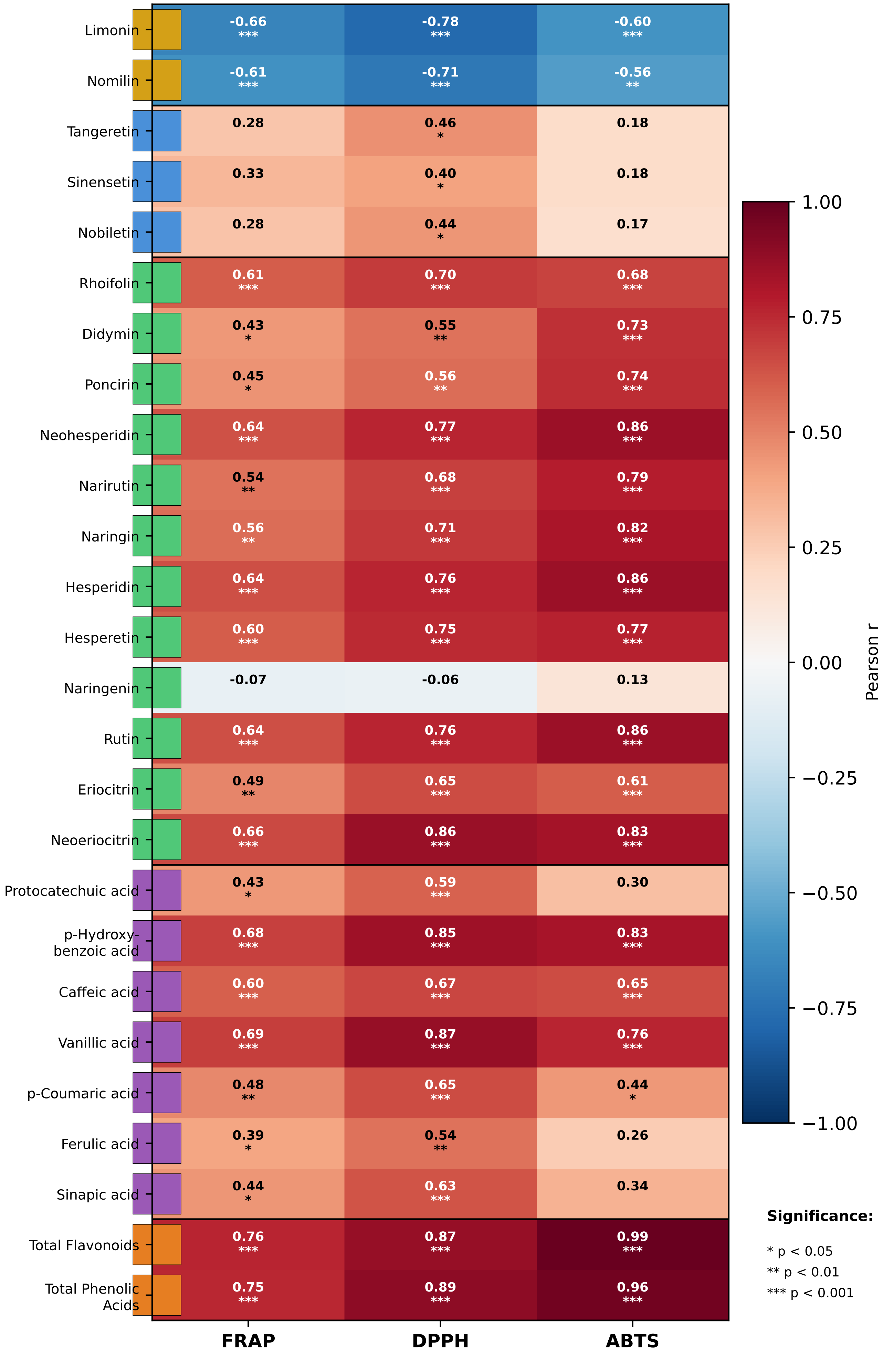

Limonin -0.66 *** -0.78 *** -0.60 ***
Nomilin -0.61 *** -0.71 *** -0.56 **
Tangeretin 0.28 0.46 * 0.18
Sinensetin 0.33 0.40 * 0.18
Nobiletin 0.28 0.44 * 0.17
Rhoifolin 0.61 *** 0.70 *** 0.68 ***
Didymin 0.43 * 0.55 ** 0.73 ***
Poncirin 0.45 * 0.56 ** 0.74 ***
Neohesperidin 0.64 *** 0.77 *** 0.86 ***
Narirutin 0.54 ** 0.68 *** 0.79 ***
Naringin 0.56 ** 0.71 *** 0.82 ***
Hesperidin 0.64 *** 0.76 *** 0.86 ***
Hesperetin 0.60 *** 0.75 *** 0.77 ***
Naringenin -0.07 -0.06 0.13
Rutin 0.64 *** 0.76 *** 0.86 ***
Eriocitrin 0.49 ** 0.65 *** 0.61 ***
Neoeriocitrin 0.66 *** 0.86 *** 0.83 ***
Protocatechuic acid 0.43 * 0.59 *** 0.30
p-Hydroxy-benzoic acid 0.68 *** 0.85 *** 0.83 ***
Caffeic acid 0.60 *** 0.67 *** 0.65 ***
Vanillic acid 0.69 *** 0.87 *** 0.76 ***
p-Coumaric acid 0.48 ** 0.65 *** 0.44 *
Ferulic acid 0.39 * 0.54 ** 0.26
Sinapic acid 0.44 * 0.63 *** 0.34
Total Flavonoids 0.76 *** 0.87 *** 0.99 ***
Total Phenolic Acids 0.75 *** 0.89 *** 0.96 ***
FRAP
DPPH
ABTS
1.00
0.75
0.50
0.25
0.00
−0.25
−0.50
−0.75
−1.00
Pearson r
Significance:
* p < 0.05
** p < 0.01
*** p < 0.001
