# Supplementary material for: Tissue-Specific Phytochemical Profiles of Two Huyou Cultivars: Bioactive Compounds and Thermal Processing Effects
Source: Foods. 2026 Oct 23;15(21):3805. doi: 10.3390/foods15213805 (PMC13762711; doi:10.3390/foods15213805)
Supplement: Supplementary file 1 [file foods-15-03805-s001.zip › Figure S3.pdf]

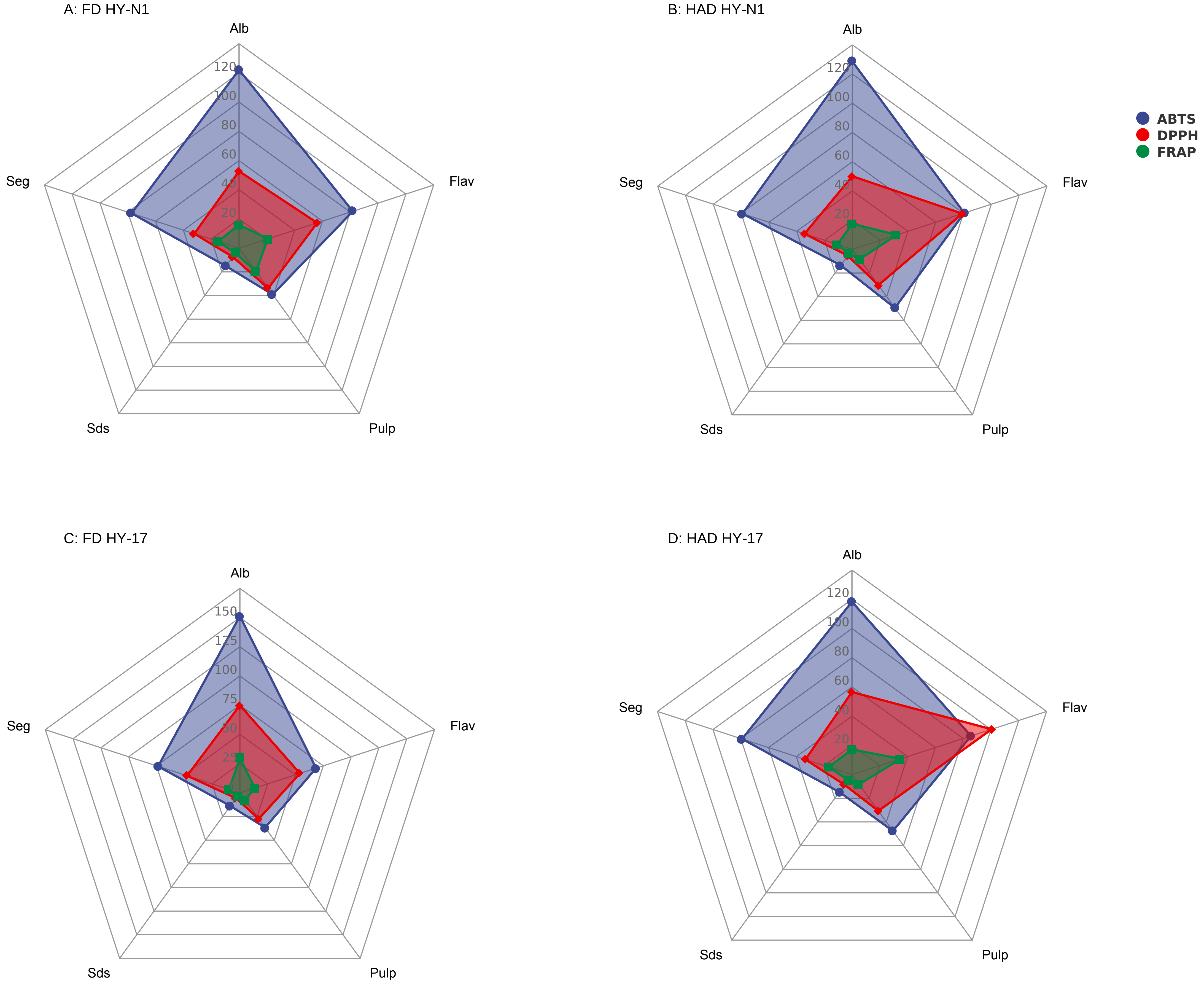

A: FD HY-N1
B: HAD HY-N1
C: FD HY-17
D: HAD HY-17
Alb
Flav
Pulp
Sds
Seg
120
100
80
60
40
20
150
125
100
75
50
25
ABTS
DPPH
FRAP
