# Supplementary material for: Tissue-Specific Phytochemical Profiles of Two Huyou Cultivars: Bioactive Compounds and Thermal Processing Effects
Source: Foods. 2026 Oct 23;15(21):3805. doi: 10.3390/foods15213805 (PMC13762711; doi:10.3390/foods15213805)
Supplement: Supplementary file 1 [file foods-15-03805-s001.zip › Supplementary Tables.pdf]

## Supplementary Materials:

**Table S1. Chromatographic conditions for analysis of soluble sugars, organic acids, and free amino acids.**

| Items | Steps |
|---|---|
| Soluble sugars (sucrose, glucose, fructose) and organic acids (9 acids) | For sugar analysis, approximately 1 g of freeze-dried pulp sample was accurately weighed, diluted with pure water to a final volume of 50 mL, and filtered through a 0.22 μm membrane prior to injection. For organic acid analysis, 1 g of juice sample was similarly diluted to 50 mL with water; if necessary, 50 mg of weak cation exchange (WCX) sorbent was added for adsorption purification before filtration.<br>Analysis was performed on a RIGOL L3000 HPLC system. Sugars were separated on a Rezex RCM-Monosaccharide Ca column (300 × 7.8 mm) at 63 °C, using 100% deionized water as the mobile phase at a flow rate of 0.5 mL/min, with detection by a refractive index detector (RID) at 40 °C. Organic acids were separated on a Titan C18 column at 35 °C, using a mobile phase of methanol : 40 mmol/L potassium dihydrogen phosphate buffer (pH = 2.4, adjusted with 0.1 mol/L phosphoric acid) = 2 : 98 (v/v) at a flow rate of 0.8 mL/min, with UV detection at 210 nm. |
| Free amino acids | For pre-column derivatization, 200 μL of the supernatant was mixed with 20 μL of norleucine (internal standard). Derivatization was initiated by adding 100 μL of triethylamine acetonitrile solution (pH > 7) and 100 μL of phenyl isothiocyanate (PITC) acetonitrile solution, followed by incubation at 25 °C for 1 hour. Subsequently, 400 μL of n-hexane was added for liquid-liquid extraction. After mixing and standing for 10 min, the lower aqueous layer was collected, filtered through a 0.22 μm membrane, and subjected to HPLC analysis.<br>Chromatographic separation was conducted on a RIGOL L3000 HPLC system with a UV detector. Separation was achieved using a Sepax Amethyst C18-H column (250 mm × 4.6 mm, 5 μm) maintained at 40 °C, with a flow rate of 1.0 mL/min and an injection volume of 10 μL. The mobile phase consisted of (A) sodium acetate-acetonitrile solution (7.6 g anhydrous sodium acetate dissolved in 925 mL water, pH adjusted to 6.5 with glacial acetic acid, then mixed with 70 mL acetonitrile) and (B) 80% acetonitrile aqueous solution. Detection was performed at 254 nm. |

**Table S2. Method validation parameters for analysis of soluble sugars, organic acids, and free amino acids.**

| Analyte | Methods | LOD (mg/L) | Linear range (mg/L) | Regression equation | $R^2$ |
|---|---|---|---|---|---|
| Sucrose | HPLC-RID | 2.3 | 10–500 | y = 5.003x − 0.327 | 0.9999 |
| Glucose | HPLC-RID | 2.3 | 10–500 | y = 6.499x + 1.052 | 0.9999 |
| Fructose | HPLC-RID | 2.3 | 10–500 | y = 5.229x − 0.475 | 0.9999 |
| Tartaric acid | HPLC-UV | 0.5 | 5–200 | y = 4.672x + 10.09 | 0.9997 |
| Malic acid | HPLC-UV | 0.5 | 5–200 | y = 32.57x + 555.5 | 0.9998 |
| Shikimic acid | HPLC-UV | 0.5 | 5–200 | y = 0.0094x − 0.367 | 0.9997 |
| Citric acid | HPLC-UV | 0.5 | 5–200 | y = 105.2x + 582.4 | 0.9998 |
| Quinic acid | HPLC-UV | 0.5 | 5–200 | y = 0.532x + 1.076 | 0.9996 |

| | | | | | |
|---|---|---|---|---|---|
| Lactic acid | HPLC-UV | 0.5 | 5–200 | y = 1.248x + 3.562 | 0.9995 |
| Acetic acid | HPLC-UV | 0.5 | 5–200 | y = 0.875x + 2.134 | 0.9995 |
| Succinic acid | HPLC-UV | 0.5 | 5–200 | y = 28.43x + 412.6 | 0.9998 |
| Fumaric acid | HPLC-UV | 0.1 | 1–50 | y = 156.7x + 28.13 | 0.9999 |
| Aspartic acid | HPLC-UV | 0.12 | 1–200 | y = 4.202x + 1.053 | 0.9999 |
| Glutamic acid | HPLC-UV | 0.14 | 1–200 | y = 4.746x + 2.467 | 0.9999 |
| Serine | HPLC-UV | 0.10 | 1–200 | y = 9.872x + 1.526 | 0.9999 |
| Glycine | HPLC-UV | 0.08 | 1–200 | y = 10.648x + 0.997 | 0.9999 |
| Histidine | HPLC-UV | 0.15 | 1–200 | y = 10.944x + 1.243 | 0.9999 |
| Arginine | HPLC-UV | 0.12 | 1–200 | y = 12.836x + 0.876 | 0.9999 |
| Threonine | HPLC-UV | 0.10 | 1–200 | y = 13.991x + 1.105 | 0.9999 |
| Alanine | HPLC-UV | 0.08 | 1–200 | y = 14.661x + 0.712 | 0.9999 |
| Proline | HPLC-UV | 0.20 | 1–200 | y = 15.282x + 1.634 | 0.9999 |
| Tyrosine | HPLC-UV | 0.18 | 1–200 | y = 22.360x + 1.087 | 0.9999 |
| Valine | HPLC-UV | 0.10 | 1–200 | y = 23.892x + 0.955 | 0.9999 |
| Methionine | HPLC-UV | 0.15 | 1–200 | y = 24.927x + 1.312 | 0.9999 |
| Isoleucine | HPLC-UV | 0.10 | 1–200 | y = 26.213x + 0.845 | 0.9999 |
| Leucine | HPLC-UV | 0.10 | 1–200 | y = 27.057x + 1.168 | 0.9999 |
| Phenylalanine | HPLC-UV | 0.12 | 1–200 | y = 27.415x + 0.723 | 0.9999 |
| Lysine | HPLC-UV | 0.15 | 1–200 | y = 28.996x + 1.446 | 0.9999 |

**Table S3. Extraction procedures and instrumental parameters for analysis of limonoids, flavonoids, and phenolic acids.**

| Items | Steps |
|---|---|
| Limonoids (limonin and nomilin) | Limonoids were extracted from approximately 0.10 g of sample with 1.0 mL of 80% methanol via ultrasonication for 1 h, followed by centrifugation. The supernatant was filtered through a 0.22 μm membrane and analyzed using an Agilent 1100 HPLC system equipped with a Compass C18 column (250 mm × 4.6 mm, 5 μm) at 30 °C. The mobile phase consisted of acetonitrile/water at a flow rate of 1.0 mL/min, with the ratio adjusted (40:60 or 50:50, v/v) to resolve interference peaks. Detection was performed at 210 nm, and quantification was based on external standard calibration curves. |
| Flavonoids (flavanone glycosides, PMFs) | Following extraction with acetonitrile containing 1% acetic acid and QuEChERS purification, flavonoids were analyzed using a SCIEX Triple Quad 5500+ UPLC-MS/MS system. Chromatographic separation was performed on a Kinetex C18 column (3.0 × 150 mm, 2.6 μm) at 40 °C, using a gradient elution of methanol and 5 mM ammonium acetate in water at a flow rate of 0.30 mL/min. The injection volume was 1.0 μL. Mass spectrometric detection employed electrospray ionization in positive mode ($ESI^+$) under multiple reaction monitoring (MRM), with a capillary temperature of 350 °C and a spray voltage of 4500 V. |
| Phenolic acids (7 compounds) | Phenolic acids were extracted following alkaline hydrolysis and liquid-liquid extraction, then determined using a Waters ACQUITY UPLC system (2996 PDA detector) with a BEH C18 column (2.1 mm × 100 mm, 1.7 μm). The mobile phase comprised 3.0% formic acid in water (A) |

and methanol (B) under gradient elution at a flow rate of 0.30 mL/min, with a column temperature of 35 °C and an injection volume of 3.0 μL. Detection wavelengths were set at 260 nm for protocatechuic acid, p-hydroxybenzoic acid, and vanillic acid, and 320 nm for caffeic acid, p-coumaric acid, sinapic acid, and ferulic acid. Compounds were identified based on retention times and UV-Vis spectra, with quantification performed using the external standard method.

**Table S4. Method validation parameters for analysis of limonoids, flavonoids, and phenolic acids.**

| Analyte | Detection method | LOD | Linear range | Regression equation | $R^2$ |
|---|---|---|---|---|---|
| Limonin | HPLC-UV | 0.10 mg/L | 0.4–500 mg/L | y = 5.003x − 0.327 | 0.9999 |
| Nomilin | HPLC-UV | 0.12 mg/L | 0.4–500 mg/L | y = 6.499x + 1.052 | 0.9999 |
| Obacunone | HPLC-UV | 0.015 mg/L | 0.04–40 mg/L | y = 23.671x − 0.600 | 0.9999 |
| Tangeretin | UPLC-MS/MS | 5 μg/kg | 1–1000 μg/kg | y = 44161x + 186007 | 0.9965 |
| Sinensetin | UPLC-MS/MS | 5 μg/kg | 1–1000 μg/kg | y = 5108x + 14304 | 0.9935 |
| Rhoifolin | UPLC-MS/MS | 5 μg/kg | 1–1000 μg/kg | y = 694x + 1644 | 0.9970 |
| Nobiletin | UPLC-MS/MS | 5 μg/kg | 1–1000 μg/kg | y = 12868x + 69801 | 0.9902 |
| Spinosin | UPLC-MS/MS | 5 μg/kg | 1–1000 μg/kg | y = 44x + 559 | 0.9971 |
| Didymin | UPLC-MS/MS | 5 μg/kg | 1–1000 μg/kg | y = 297x + 1951 | 0.9986 |
| Poncirin | UPLC-MS/MS | 5 μg/kg | 1–1000 μg/kg | y = 319x + 2173 | 0.9982 |
| Neohesperidin | UPLC-MS/MS | 5 μg/kg | 1–1000 μg/kg | y = 184x + 14823 | 0.9968 |
| Narirutin | UPLC-MS/MS | 5 μg/kg | 1–1000 μg/kg | y = 2569x + 108179 | 0.9979 |
| Naringin | UPLC-MS/MS | 5 μg/kg | 1–1000 μg/kg | y = 693x + 8612 | 0.9950 |
| Hesperidin | UPLC-MS/MS | 5 μg/kg | 1–1000 μg/kg | y = 117x + 10018 | 0.9963 |
| Hesperetin | UPLC-MS/MS | 5 μg/kg | 1–1000 μg/kg | y = 3928x + 6616 | 0.9980 |
| Naringenin | UPLC-MS/MS | 5 μg/kg | 1–1000 μg/kg | y = 8057x + 19827 | 0.9937 |
| Phloretin | UPLC-MS/MS | 5 μg/kg | 1–1000 μg/kg | y = 31912x + 57756 | 0.9939 |
| Vitexin | UPLC-MS/MS | 5 μg/kg | 1–1000 μg/kg | y = 8181x + 252 | 0.9971 |
| Eriocitrin | UPLC-MS/MS | 5 μg/kg | 1–1000 μg/kg | y = 2584x + 12932 | 0.9950 |
| Rutin | UPLC-MS/MS | 5 μg/kg | 1–1000 μg/kg | y = 809x + 22674 | 0.9954 |
| Protocatechuic acid | UPLC-PDA | 0.10 mg/L | 0.5–100 mg/L | y = 2.855x + 1.563 | 0.9998 |
| p-Hydroxybenzoic acid | UPLC-PDA | 0.08 mg/L | 0.5–100 mg/L | y = 3.245x + 0.872 | 0.9999 |
| Vanillic acid | UPLC-PDA | 0.10 mg/L | 0.5–100 mg/L | y = 2.968x + 1.201 | 0.9999 |
| Caffeic acid | UPLC-PDA | 0.08 mg/L | 0.5–100 mg/L | y = 3.512x + 0.654 | 0.9998 |
| p-Coumaric acid | UPLC-PDA | 0.06 mg/L | 0.5–100 mg/L | y = 4.103x + 1.108 | 0.9999 |

| | | | | | |
|---|---|---|---|---|---|
| Sinapic acid | UPLC-PDA | 0.10 mg/L | 0.5–100 mg/L | $y = 3.876x + 0.935$ | 0.9998 |
| Ferulic acid | UPLC-PDA | 0.06 mg/L | 0.5–100 mg/L | $y = 4.225x + 0.786$ | 0.9999 |

**Table S5. Antioxidant assay protocols for DPPH, ABTS, and FRAP methods.**

| Method | Protocol |
|---|---|
| DPPH assay | The DPPH (2,2-diphenyl-1-picrylhydrazyl) radical scavenging activity was determined using a commercial kit (Suzhou Mengxi Biomedical Technology Co., Ltd., China). Twenty microliters of the sample supernatant was mixed with 380 µL of DPPH working solution. The mixture was thoroughly vortexed and incubated in the dark at room temperature for 30 min. After incubation, 200 µL of the reaction mixture was transferred to a 96-well plate, and the absorbance was measured at 515 nm using a microplate reader. The decrease in absorbance, calculated as $\Delta A = A\_blank - A\_sample$, was proportional to the radical scavenging activity. The standard curve was constructed using Trolox standard solutions ($y = 0.5324x - 0.0069$, $R^2 = 0.9997$, where $y = \Delta A$ and $x$ = Trolox concentration in µmol/mL). The DPPH radical scavenging capacity was expressed as µmol Trolox equivalents per gram of dry weight (µmol TE/g DW), calculated as $TEAC = 1.878 \times (\Delta A + 0.0069) \div W$, where W is the sample weight in grams. |
| ABTS assay | The ABTS [2,2′-azino-bis(3-ethylbenzothiazoline-6-sulfonic acid)] radical cation scavenging activity was measured using a commercial kit (Suzhou Mengxi Biomedical Technology Co., Ltd., China). Ten microliters of the sample supernatant was mixed with 190 µL of ABTS working solution. The reaction mixture was incubated at room temperature for 6 min, and the absorbance was immediately measured at 734 nm using a microplate reader. The decrease in absorbance, expressed as $\Delta A = A\_blank - A\_sample$, reflected the antioxidant capacity. The standard curve followed $y = 0.55x + 0.0006$ ($R^2 = 0.9992$), where $y = \Delta A$ and $x$ = Trolox concentration in µmol/mL. The ABTS radical scavenging capacity was calculated and expressed as µmol TE/g DW using the formula: $TEAC = 1.818 \times (\Delta A - 0.0006) \div W$. |
| FRAP assay | The ferric reducing antioxidant power (FRAP) was evaluated using a commercial kit (Suzhou Mengxi Biomedical Technology Co., Ltd., China). The FRAP working solution was freshly prepared by mixing reagent 1, reagent 2, and reagent 3 at a ratio of 10:1:1 (v/v/v) and pre-warmed to 37 °C. Ten microliters of the sample supernatant was mixed with 190 µL of the pre-warmed FRAP working solution and incubated at 37 °C for 10 min. The absorbance was measured at 593 nm, and the absorbance increase was calculated as $\Delta A = A\_sample - A\_blank$. The standard curve equation was $y = 1.2994x - 0.002$ ($R^2 = 1.0000$), where $y = \Delta A$ and $x$ = Trolox concentration in µmol/mL. Results were expressed as µmol TE/g DW, calculated as $TEAC = 0.7696 \times (\Delta A + 0.002) \div W$. |

**Table S6. Comparative chemical profile of ‘Changhu No. 1’ and ‘Huyou 01-7’.**

| Parameter | Changhu No. 1 (HY-N1) | Huyou 01-7 (HY-17) | P-value |
|---|---|---|---|
| Single fruit weight (g) | 279.85 ± 24.26 a | 242.80 ± 25.95 a | ns |
| Transverse diameter (mm) | 85.10 ± 3.56 a | 82.24 ± 3.22 a | ns |
| Longitudinal diameter (mm) | 75.86 ± 2.74 a | 70.19 ± 3.35 a | ns |
| Pericarp thickness (mm) | 6.97 ± 0.71 a | 5.69 ± 0.43 b | HY-N1 > HY-17 |
| Fruit shape index | 0.89 ± 0.01 a | 0.85 ± 0.01 b | HY-N1 > HY-17 |
| Edible rate (%) | 64.54 ± 1.98 a | 67.66 ± 0.34 a | ns |
| Juice yield (%) | 35.49 ± 1.09 b | 41.27 ± 0.21 a | HY-17 > HY-N1 |
| L* | 72.02 ± 0.22 a | 74.10 ± 1.94 a | ns |
| a* | 11.86 ± 1.71 a | 11.14 ± 1.91 a | ns |
| b* | 66.84 ± 0.70 a | 67.18 ± 0.93 a | ns |
| ΔE*ab | 61.95 ± 0.44 a | 63.03 ± 1.41 a | ns |
| TSS (%) | 15.23 ± 0.55 a | 14.67 ± 0.15 a | ns |
| TA (g/100 mL) | 1.49 ± 0.04 a | 1.16 ± 0.05 b | HY-N1 > HY-17 |
| Vitamin C (mg/100 g) | 44.90 ± 1.06 a | 37.17 ± 1.33 b | HY-N1 > HY-17 |
| Total sugar (%) | 12.07 ± 0.67 a | 10.40 ± 0.10 b | HY-N1 > HY-17 |
| Reducing sugar (%) | 5.47 ± 0.40 a | 4.73 ± 0.12 b | HY-N1 > HY-17 |
| Sucrose (%) | 6.27 ± 0.40 a | 5.37 ± 0.06 b | HY-N1 > HY-17 |
| TSS/TA ratio | 10.21 ± 0.53 b | 12.69 ± 0.47 a | HY-17 > HY-N1 |
| Sugar/acid ratio | 8.09 ± 0.59 a | 9.00 ± 0.32 a | ns |
| Sucrose (mg/g DW) | 387.24 ± 28.55 a | 407.00 ± 27.96 a | ns |
| Glucose (mg/g DW) | 152.79 ± 11.29 a | 157.23 ± 10.08 a | ns |
| Fructose (mg/g DW) | 161.09 ± 10.87 a | 165.66 ± 11.14 a | ns |
| Total sugar (mg/g DW) | 701.12 ± 49.35 a | 729.89 ± 48.73 a | ns |
| Tartaric acid (mg/g DW) | 8.39 ± 0.97 a | 7.32 ± 0.40 a | ns |
| Malic acid (mg/g DW) | 12.60 ± 0.09 a | 11.14 ± 1.33 a | ns |
| Shikimic acid (mg/g DW) | 0.51 ± 0.01 a | 0.48 ± 0.03 a | ns |

| | | | |
|---|---|---|---|
| Citric acid (mg/g DW) | 75.68 ± 1.42 a | 68.17 ± 4.68 b | HY-N1 > HY-17 |
| Succinic acid (mg/g DW) | ND | ND | |
| Oxalic acid | ND | ND | |
| Lactic acid | ND | ND | |
| Fumaric acid | ND | ND | |
| Acetic acid | ND | ND | |
| Total organic acids (mg/g DW) | 96.67 ± 2.29 a | 97.17 ± 2.29 a | ns |
| Thr (Threonine, mg/kg DW) | 161.58 ± 9.47 a | 146.17 ± 6.82 a | ns |
| Lys (Lysine, mg/kg DW) | 124.76 ± 10.24 a | 133.53 ± 2.13 a | ns |
| Val (Valine, mg/kg DW) | 94.03 ± 2.67 a | 76.27 ± 3.23 b | HY-N1 > HY-17 |
| Phe (Phenylalanine, mg/kg DW) | 66.29 ± 1.27 a | 56.27 ± 4.66 b | HY-N1 > HY-17 |
| Ile (Isoleucine, mg/kg DW) | 49.44 ± 0.77 a | 41.74 ± 1.39 b | HY-N1 > HY-17 |
| Leu (Leucine, mg/kg DW) | 30.73 ± 0.95 a | 31.90 ± 2.00 a | ns |
| Met (Methionine, mg/kg DW) | 19.28 ± 0.89 a | 18.78 ± 0.68 a | ns |
| Arg (Arginine, mg/kg DW) | 6613.14 ± 525.56 b | 7605.60 ± 92.88 a | HY-17 > HY-N1 |
| His (Histidine, mg/kg DW) | 67.58 ± 5.37 a | 68.84 ± 1.99 a | ns |
| Pro (Proline, mg/kg DW) | 7617.25 ± 1144.71 a | 3370.99 ± 112.25 b | HY-N1 > HY-17 |
| Asp (Aspartic acid, mg/kg DW) | 4460.50 ± 204.76 b | 5372.43 ± 220.80 a | HY-17 > HY-N1 |
| Glu (Glutamic acid, mg/kg DW) | 1204.90 ± 129.47 a | 1382.84 ± 27.66 a | ns |
| Ser (Serine, mg/kg DW) | 1160.02 ± 62.22 a | 1044.73 ± 14.92 b | HY-N1 > HY-17 |
| Ala (Alanine, mg/kg DW) | 1070.60 ± 58.27 a | 882.06 ± 49.38 b | HY-N1 > HY-17 |
| Gly (Glycine, mg/kg DW) | 124.22 ± 7.43 a | 103.03 ± 0.78 b | HY-N1 > HY-17 |
| Tyr (Tyrosine, mg/kg DW) | 26.70 ± 1.21 a | 23.32 ± 0.80 b | HY-N1 > HY-17 |
| Cys (Cystine, mg/kg DW) | ND | ND | |
| Total FAA (mg/kg DW) | 22891.01 ± 1265.56 a | 20358.59 ± 58.91 b | HY-N1 > HY-17 |

**Table S7. Tissue-specific distribution of secondary metabolites in Huyou cultivars (mg/kg DW).**

| Metabolite Class | Compound | Tissue | Changhu No. 1 (HY-N1) | Huyou 01-7 (HY-17) | P-value |
|---|---|---|---|---|---|
| Bitter Limonoids | Limonin | Pulp | 339.44±5.55 | 267.93±21.09 | HY-N1 > HY-17 (P=0.022) |
| Bitter Limonoids | Limonin | Flav | 339.99±106.87 | 72.60±5.44 | HY-N1 > HY-17 (P=0.049) |
| Bitter Limonoids | Limonin | Alb | 145.37±16.92 | 89.66±31.05 | ns (P=0.070) |
| Bitter Limonoids | Limonin | Seg | 1210.53±73.40 | 1177.21±119.01 | ns (P=0.705) |
| Bitter Limonoids | Limonin | Sds | 8437.65±208.01 | 8751.99±161.72 | ns (P=0.241) |
| Bitter Limonoids | Nomilin | Pulp | 20.67±0.02 | 11.33±0.76 | HY-N1 > HY-17 (P=0.002) |
| Bitter Limonoids | Nomilin | Flav | 115.48±16.41 | 123.18±6.61 | ns (P=0.512) |
| Bitter Limonoids | Nomilin | Alb | 7.77±2.24 | 12.34±0.79 | ns (P=0.058) |
| Bitter Limonoids | Nomilin | Seg | 95.27±68.30 | 71.52±27.82 | ns (P=0.621) |
| Bitter Limonoids | Nomilin | Sds | 2444.77±20.43 | 1434.40±35.07 | HY-N1 > HY-17 (P=0.003) |
| Flavonoids | Tangeretin | Pulp | 1.89±1.08 | 2.39±0.71 | ns (P=0.368) |
| Flavonoids | Tangeretin | Flav | 546.68±78.42 | 564.26±23.47 | ns (P=0.618) |
| Flavonoids | Tangeretin | Alb | 45.56±15.88 | 81.17±6.19 | HY-N1 < HY-17 (P=0.002) |
| Flavonoids | Tangeretin | Seg | 6.25±2.20 | 10.06±2.13 | HY-N1 < HY-17 (P=0.012) |
| Flavonoids | Tangeretin | Sds | 1.79±0.45 | 3.44±0.47 | HY-N1 < HY-17 (P<0.001) |
| Flavonoids | Sinensetin | Pulp | 0.135±0.060 | 0.0433±0.0186 | HY-N1 > HY-17 (P=0.012) |
| Flavonoids | Sinensetin | Flav | 18.39±1.49 | 6.37±0.47 | HY-N1 > HY-17 (P<0.001) |
| Flavonoids | Sinensetin | Alb | 1.69±0.50 | 1.10±0.19 | HY-N1 > HY-17 (P=0.033) |
| Flavonoids | Sinensetin | Seg | 0.250±0.095 | 0.182±0.065 | ns (P=0.182) |
| Flavonoids | Sinensetin | Sds | 0.0850±0.0055 | 0.1050±0.0055 | HY-N1 < HY-17 (P<0.001) |
| Flavonoids | Rhoifolin | Pulp | 9.83±1.00 | 6.07±0.93 | HY-N1 > HY-17 (P<0.001) |
| Flavonoids | Rhoifolin | Flav | 366.58±105.02 | 174.70±47.32 | HY-N1 > HY-17 (P=0.005) |
| Flavonoids | Rhoifolin | Alb | 484.31±35.82 | 341.55±109.90 | HY-N1 > HY-17 (P=0.023) |
| Flavonoids | Rhoifolin | Seg | 15.19±0.59 | 11.13±3.36 | HY-N1 > HY-17 (P=0.031) |
| Flavonoids | Rhoifolin | Sds | 2.85±0.32 | 3.91±0.43 | HY-N1 < HY-17 (P<0.001) |
| Flavonoids | Nobiletin | Pulp | 3.60±1.46 | 4.18±0.72 | ns (P=0.408) |

| | | | | | |
|---|---|---|---|---|---|
| Flavonoids | Nobiletin | Flav | 1629.92±175.77 | 1364.55±34.58 | HY-N1 > HY-17 (P=0.013) |
| Flavonoids | Nobiletin | Alb | 114.14±34.51 | 175.47±18.80 | HY-N1 < HY-17 (P=0.005) |
| Flavonoids | Nobiletin | Seg | 17.04±6.21 | 22.87±4.87 | ns (P=0.102) |
| Flavonoids | Nobiletin | Sds | 3.56±0.66 | 6.55±0.59 | HY-N1 < HY-17 (P<0.001) |
| Flavonoids | Didymin | Pulp | 9.50±0.46 | 5.33±0.15 | HY-N1 > HY-17 (P<0.001) |
| Flavonoids | Didymin | Flav | 13.38±5.39 | 10.35±7.30 | ns (P=0.434) |
| Flavonoids | Didymin | Alb | 163.86±9.20 | 125.82±10.05 | HY-N1 > HY-17 (P<0.001) |
| Flavonoids | Didymin | Seg | 115.87±24.42 | 94.23±10.43 | ns (P=0.087) |
| Flavonoids | Didymin | Sds | 0.775±0.097 | 1.02±0.10 | HY-N1 < HY-17 (P=0.002) |
| Flavonoids | Poncirin | Pulp | 10.79±0.60 | 5.97±0.24 | HY-N1 > HY-17 (P<0.001) |
| Flavonoids | Poncirin | Flav | 14.61±6.01 | 11.85±7.96 | ns (P=0.514) |
| Flavonoids | Poncirin | Alb | 174.31±8.46 | 139.06±12.38 | HY-N1 > HY-17 (P<0.001) |
| Flavonoids | Poncirin | Seg | 127.21±26.22 | 103.25±10.67 | ns (P=0.079) |
| Flavonoids | Poncirin | Sds | 0.855±0.046 | 1.11±0.09 | HY-N1 < HY-17 (P<0.001) |
| Flavonoids | Neohesperidin | Pulp | 803.58±81.50 | 689.88±20.33 | HY-N1 > HY-17 (P=0.018) |
| Flavonoids | Neohesperidin | Flav | 3670.97±737.84 | 2520.11±653.60 | HY-N1 > HY-17 (P=0.017) |
| Flavonoids | Neohesperidin | Alb | 8610.68±273.66 | 7961.02±422.59 | HY-N1 > HY-17 (P=0.012) |
| Flavonoids | Neohesperidin | Seg | 4380.34±406.20 | 3911.98±353.23 | ns (P=0.059) |
| Flavonoids | Neohesperidin | Sds | 45.84±1.92 | 77.18±7.96 | HY-N1 < HY-17 (P<0.001) |
| Flavonoids | Narirutin | Pulp | 1914.77±138.92 | 2041.32±226.02 | ns (P=0.275) |
| Flavonoids | Narirutin | Flav | 2337.68±205.93 | 2170.08±351.93 | ns (P=0.343) |
| Flavonoids | Narirutin | Alb | 7208.49±363.30 | 6851.96±268.01 | ns (P=0.084) |
| Flavonoids | Narirutin | Seg | 5718.76±130.25 | 5962.30±255.74 | ns (P=0.074) |
| Flavonoids | Narirutin | Sds | 117.04±3.37 | 142.66±10.41 | HY-N1 < HY-17 (P=0.001) |
| Flavonoids | Naringin | Pulp | 2336.05±222.94 | 2297.51±216.30 | ns (P=0.767) |
| Flavonoids | Naringin | Flav | 4400.36±457.91 | 3988.16±694.95 | ns (P=0.257) |
| Flavonoids | Naringin | Alb | 13580.56±721.29 | 12686.32±457.65 | HY-N1 > HY-17 (P=0.032) |
| Flavonoids | Naringin | Seg | 9444.41±282.95 | 9596.30±649.85 | ns (P=0.616) |
| Flavonoids | Naringin | Sds | 144.01±3.13 | 191.50±15.66 | HY-N1 < HY-17 (P<0.001) |

| | | | | | |
|---|---|---|---|---|---|
| Flavonoids | Hesperidin | Pulp | 624.24±53.38 | 557.32±25.38 | HY-N1 > HY-17 (P=0.027) |
| Flavonoids | Hesperidin | Flav | 2628.28±525.96 | 1789.25±476.17 | HY-N1 > HY-17 (P=0.016) |
| Flavonoids | Hesperidin | Alb | 6301.70±203.39 | 5884.00±358.39 | HY-N1 > HY-17 (P=0.038) |
| Flavonoids | Hesperidin | Seg | 3219.72±314.29 | 2844.28±237.89 | HY-N1 > HY-17 (P=0.044) |
| Flavonoids | Hesperidin | Sds | 35.59±2.18 | 59.78±5.60 | HY-N1 < HY-17 (P<0.001) |
| Flavonoids | Hesperetin | Pulp | n.d. | n.d. | |
| Flavonoids | Hesperetin | Flav | 0.655±0.189 | 0.518±0.190 | ns (P=0.240) |
| Flavonoids | Hesperetin | Alb | 0.788±0.095 | 1.86±1.66 | ns (P=0.173) |
| Flavonoids | Hesperetin | Seg | 0.253±0.048 | 0.470±0.210 | ns (P=0.052) |
| Flavonoids | Hesperetin | Sds | 0.125±0.023 | 0.107±0.019 | ns (P=0.166) |
| Flavonoids | Naringenin | Pulp | n.d. | n.d. | |
| Flavonoids | Naringenin | Flav | 0.902±0.279 | 1.08±0.86 | ns (P=0.640) |
| Flavonoids | Naringenin | Alb | 1.23±0.16 | 6.59±8.14 | ns (P=0.167) |
| Flavonoids | Naringenin | Seg | 0.588±0.170 | 1.65±1.54 | ns (P=0.155) |
| Flavonoids | Naringenin | Sds | 1.94±0.07 | 1.10±0.09 | HY-N1 > HY-17 (P<0.001) |
| Flavonoids | Rutin | Pulp | 655.83±62.34 | 580.80±20.77 | HY-N1 > HY-17 (P=0.031) |
| Flavonoids | Rutin | Flav | 2857.17±608.83 | 1933.26±502.47 | HY-N1 > HY-17 (P=0.017) |
| Flavonoids | Rutin | Alb | 6893.90±246.18 | 6451.03±435.98 | ns (P=0.063) |
| Flavonoids | Rutin | Seg | 3493.64±346.07 | 3081.73±241.75 | HY-N1 > HY-17 (P=0.041) |
| Flavonoids | Rutin | Sds | 35.81±2.04 | 60.91±5.87 | HY-N1 < HY-17 (P<0.001) |
| Flavonoids | Eriocitrin | Pulp | 706.59±37.77 | 767.71±82.05 | ns (P=0.141) |
| Flavonoids | Eriocitrin | Flav | 517.30±26.44 | 574.35±95.48 | ns (P=0.210) |
| Flavonoids | Eriocitrin | Alb | 820.77±138.91 | 1072.23±78.61 | HY-N1 < HY-17 (P=0.005) |
| Flavonoids | Eriocitrin | Seg | 1012.90±34.52 | 1368.91±73.66 | HY-N1 < HY-17 (P<0.001) |
| Flavonoids | Eriocitrin | Sds | 26.58±0.97 | 42.16±4.27 | HY-N1 < HY-17 (P<0.001) |
| Flavonoids | Neoeriocitrin | Pulp | 377.79±52.63 | 358.76±16.43 | ns (P=0.430) |
| Flavonoids | Neoeriocitrin | Flav | 3057.36±395.38 | 2981.51±317.16 | ns (P=0.722) |
| Flavonoids | Neoeriocitrin | Alb | 4002.37±248.80 | 4084.74±249.38 | ns (P=0.579) |
| Flavonoids | Neoeriocitrin | Seg | 1942.04±147.61 | 2099.08±233.58 | ns (P=0.199) |

| | | | | | |
|---|---|---|---|---|---|
| Flavonoids | Neoeriocitrin | Sds | 22.94±1.64 | 37.44±2.91 | HY-N1 < HY-17 (P<0.001) |
| Flavonoids | Total Flavonoids | Pulp | 7454.60±294.25 | 7317.28±326.13 | ns (P=0.236) |
| Flavonoids | Total Flavonoids | Flav | 22060.24±1284.12 | 18090.40±1275.55 | HY-N1 > HY-17 (P<0.001) |
| Flavonoids | Total Flavonoids | Alb | 48404.36±955.61 | 45863.92±927.11 | HY-N1 > HY-17 (P<0.001) |
| Flavonoids | Total Flavonoids | Seg | 29494.46±710.55 | 29108.42±887.56 | ns (P=0.200) |
| Flavonoids | Total Flavonoids | Sds | 439.79±6.18 | 628.97±22.59 | HY-17 > HY-N1 (P<0.001) |
| Phenolic Acids | Protocatechuic acid | Pulp | 4.50±0.25 | 4.66±0.23 | ns (P=0.391) |
| Phenolic Acids | Protocatechuic acid | Flav | 19.17±0.83 | 17.79±1.41 | ns (P=0.152) |
| Phenolic Acids | Protocatechuic acid | Alb | 6.10±0.29 | 5.73±0.70 | ns (P=0.391) |
| Phenolic Acids | Protocatechuic acid | Seg | 4.08±0.23 | 3.03±0.27 | HY-N1 > HY-17 (P=0.001) |
| Phenolic Acids | Protocatechuic acid | Sds | 5.29±0.09 | 5.52±0.04 | |
| Phenolic Acids | p-Hydroxybenzoic acid | Pulp | 7.50±0.45 | 6.91±0.13 | ns (P=0.077) |
| Phenolic Acids | p-Hydroxybenzoic acid | Flav | 58.76±0.21 | 56.21±4.61 | ns (P=0.349) |
| Phenolic Acids | p-Hydroxybenzoic acid | Alb | 85.01±6.60 | 76.82±4.91 | ns (P=0.097) |
| Phenolic Acids | p-Hydroxybenzoic acid | Seg | 31.82±0.45 | 28.57±1.02 | HY-N1 > HY-17 (P=0.004) |
| Phenolic Acids | p-Hydroxybenzoic acid | Sds | 9.20±0.02 | 10.70±0.02 | |
| Phenolic Acids | Caffeic acid | Pulp | 23.85±0.42 | 22.58±0.80 | HY-N1 > HY-17 (P=0.043) |
| Phenolic Acids | Caffeic acid | Flav | 38.74±2.86 | 20.78±1.68 | HY-N1 > HY-17 (P<0.001) |
| Phenolic Acids | Caffeic acid | Alb | 61.69±10.75 | 36.29±14.38 | HY-N1 > HY-17 (P=0.033) |
| Phenolic Acids | Caffeic acid | Seg | 27.72±6.64 | 20.61±1.45 | ns (P=0.120) |
| Phenolic Acids | Caffeic acid | Sds | 21.51±0.28 | 24.19±0.10 | |
| Phenolic Acids | Vanillic acid | Pulp | 35.95±2.10 | 35.34±0.17 | ns (P=0.604) |
| Phenolic Acids | Vanillic acid | Flav | 167.25±5.16 | 153.45±5.16 | HY-N1 > HY-17 (P=0.009) |
| Phenolic Acids | Vanillic acid | Alb | 162.48±2.57 | 155.70±6.26 | ns (P=0.116) |
| Phenolic Acids | Vanillic acid | Seg | 74.86±5.40 | 70.42±0.50 | ns (P=0.198) |
| Phenolic Acids | Vanillic acid | Sds | 28.26±0.77 | 30.41±0.43 | |
| Phenolic Acids | p-Coumaric acid | Pulp | 14.98±0.45 | 15.32±0.88 | ns (P=0.527) |
| Phenolic Acids | p-Coumaric acid | Flav | 128.33±8.80 | 91.93±1.55 | HY-N1 > HY-17 (P=0.003) |
| Phenolic Acids | p-Coumaric acid | Alb | 57.48±4.81 | 42.99±5.72 | HY-N1 > HY-17 (P=0.009) |

| | | | | | |
|---|---|---|---|---|---|
| Phenolic Acids | p-Coumaric acid | Seg | 35.44±3.90 | 28.39±0.28 | HY-N1 > HY-17 (P=0.036) |
| Phenolic Acids | p-Coumaric acid | Sds | 94.53±0.89 | 105.61±0.30 | |
| Phenolic Acids | Ferulic acid | Pulp | 173.20±4.37 | 182.94±3.30 | HY-N1 < HY-17 (P=0.014) |
| Phenolic Acids | Ferulic acid | Flav | 1208.93±38.07 | 976.59±18.49 | HY-N1 > HY-17 (P<0.001) |
| Phenolic Acids | Ferulic acid | Alb | 259.80±3.70 | 293.43±56.26 | ns (P=0.318) |
| Phenolic Acids | Ferulic acid | Seg | 152.52±8.63 | 140.12±8.50 | ns (P=0.087) |
| Phenolic Acids | Ferulic acid | Sds | 105.79±1.08 | 128.29±0.45 | |
| Phenolic Acids | Sinapic acid | Pulp | 61.46±1.04 | 56.40±1.25 | HY-N1 > HY-17 (P<0.001) |
| Phenolic Acids | Sinapic acid | Flav | 349.38±21.07 | 329.50±25.51 | ns (P=0.276) |
| Phenolic Acids | Sinapic acid | Alb | 110.25±5.99 | 126.78±17.63 | ns (P=0.156) |
| Phenolic Acids | Sinapic acid | Seg | 91.80±1.75 | 83.03±12.97 | ns (P=0.270) |
| Phenolic Acids | Sinapic acid | Sds | 17.40±0.09 | 23.43±0.01 | |
| Phenolic Acids | Total Phenolic Acids | Pulp | 321.44±2.25 | 324.15±2.16 | |
| Phenolic Acids | Total Phenolic Acids | Flav | 1970.58±34.41 | 1646.24±18.63 | |
| Phenolic Acids | Total Phenolic Acids | Alb | 742.81±1.95 | 737.75±105.76 | |
| Phenolic Acids | Total Phenolic Acids | Seg | 418.24±26.28 | 374.17±2.39 | |
| Phenolic Acids | Total Phenolic Acids | Sds | 281.97±3.01 | 328.14±0.68 | |

**Table S8. Tissue-specific distribution of total flavonoids, total phenolic acids, and antioxidant capacity in Huyou cultivars.**

| Metabolite Class | Assay / Compound | Tissue | Changhu No. 1 (HY-N1) | Huyou 01-7 (HY-17) | P-value |
|---|---|---|---|---|---|
| | Total Flavonoids | Pulp | 1.65±0.10 | 1.65±0.03 | ns (P=0.989) |
| | Total Flavonoids | Flav | 6.88±0.51 | 6.15±0.75 | ns (P=0.243) |
| | Total Flavonoids | Alb | 11.13±0.19 | 13.63±5.61 | ns (P=0.521) |
| | Total Flavonoids | Seg | 6.12±0.42 | 5.81±0.27 | ns (P=0.346) |
| | Total Flavonoids | Sds | 1.45±0.02 | 1.64±0.01 | HY-17 > HY-N1 (P=0.026) |
| | Total Phenolic Acids | Pulp | 7.30±0.21 | 6.78±0.10 | HY-N1 > HY-17 (P=0.032) |
| | Total Phenolic Acids | Flav | 20.05±0.99 | 18.55±2.41 | ns (P=0.401) |
| | Total Phenolic Acids | Alb | 28.72±0.39 | 28.96±9.13 | ns (P=0.968) |
| | Total Phenolic Acids | Seg | 15.68±0.61 | 14.87±1.03 | ns (P=0.321) |
| | Total Phenolic Acids | Sds | 2.34±0.02 | 2.32±0.02 | ns (P=0.349) |
| Total Antioxidant Capacity | FRAP (μmol Trolox/g) | Pulp | 19.79±0.24 | 7.62±1.77 | HY-N1 > HY-17 (P=0.006) |
| Total Antioxidant Capacity | FRAP (μmol Trolox/g) | Flav | 20.67±2.00 | 13.60±2.33 | HY-N1 > HY-17 (P=0.017) |
| Total Antioxidant Capacity | FRAP (μmol Trolox/g) | Alb | 16.12±1.54 | 29.73±6.12 | ns (P=0.054) |
| Total Antioxidant Capacity | FRAP (μmol Trolox/g) | Seg | 15.13±0.28 | 9.75±0.91 | HY-N1 > HY-17 (P=0.006) |
| Total Antioxidant Capacity | FRAP (μmol Trolox/g) | Sds | 4.15±0.32 | 4.00±0.19 | ns (P=0.651) |
| Total Antioxidant Capacity | DPPH (μmol Trolox/g) | Pulp | 33.97±3.93 | 27.51±2.71 | ns (P=0.087) |
| Total Antioxidant Capacity | DPPH (μmol Trolox/g) | Flav | 56.27±3.73 | 53.98±2.81 | ns (P=0.447) |
| Total Antioxidant Capacity | DPPH (μmol Trolox/g) | Alb | 52.73±0.33 | 74.92±18.61 | ns (P=0.175) |
| Total Antioxidant Capacity | DPPH (μmol Trolox/g) | Seg | 32.18±7.20 | 47.45±6.20 | ns (P=0.051) |
| Total Antioxidant Capacity | DPPH (μmol Trolox/g) | Sds | 7.29±0.00 | 5.66±0.00 | HY-N1 > HY-17 (P<0.001) |
| Total Antioxidant Capacity | ABTS (μmol Trolox/g) | Pulp | 39.14±0.71 | 36.72±3.06 | ns (P=0.302) |
| Total Antioxidant Capacity | ABTS (μmol Trolox/g) | Flav | 81.86±4.10 | 68.53±18.50 | ns (P=0.337) |
| Total Antioxidant Capacity | ABTS (μmol Trolox/g) | Alb | 122.14±10.64 | 151.27±68.76 | ns (P=0.541) |
| Total Antioxidant Capacity | ABTS (μmol Trolox/g) | Seg | 77.35±4.89 | 73.57±3.73 | ns (P=0.352) |
| Total Antioxidant Capacity | ABTS (μmol Trolox/g) | Sds | 15.15±0.00 | 13.87±0.00 | HY-N1 > HY-17 (P<0.001) |

**Table S9. Effect of drying methods on secondary metabolites in Huyou cultivars.**

| Metabolite Class | Compound | Tissue | HY-N1 Freeze-dried | HY-N1 Hot-air dried | HY-N1 Ret. (%) | HY-17 Freeze-dried | HY-17 Hot-air dried | HY-17 Ret. (%) |
|---|---|---|---|---|---|---|---|---|
| Amino Acid | Threonine | Pulp | 161.58 ± 9.47 a | 118.64 ± 14.50 b | 73.4 | 146.17 ± 6.82 a | 126.04 ± 8.26 b | 86.2 |
| | Lysine | Pulp | 124.76 ± 10.24 a | 47.93 ± 9.14 b | 38.4 | 133.53 ± 2.13 a | 64.02 ± 7.89 b | 47.9 |
| | Valine | Pulp | 94.03 ± 2.67 a | 48.30 ± 6.60 b | 51.4 | 76.27 ± 3.23 a | 48.02 ± 2.54 b | 63 |
| | Phenylalanine | Pulp | 66.29 ± 1.27 a | 45.61 ± 4.43 b | 68.8 | 56.27 ± 4.66 a | 52.85 ± 5.78 a | 94 |
| | Isoleucine | Pulp | 49.44 ± 0.77 a | 40.51 ± 2.77 b | 82 | 41.74 ± 1.39 b | 50.82 ± 0.94 a | 121.7 |
| | Leucine | Pulp | 30.73 ± 0.95 a | 29.06 ± 4.77 a | 94.6 | 31.90 ± 2.00 a | 44.65 ± 11.34 a | 140 |
| | Methionine | Pulp | 19.28 ± 0.89 a | 6.50 ± 3.87 b | 33.7 | 18.78 ± 0.68 a | 12.15 ± 6.00 a | 64.7 |
| | Arginine | Pulp | 6613.14 ± 525.56 a | 5149.51 ± 323.43 b | 77.9 | 7605.60 ± 92.88 a | 5976.60 ± 579.14 b | 78.6 |
| | Histidine | Pulp | 67.58 ± 5.37 a | 38.48 ± 6.67 b | 56.9 | 68.84 ± 1.99 a | 58.70 ± 5.01 a | 85.2 |
| | Proline | Pulp | 7617.25 ± 1144.71 a | 7806.87 ± 1602.72 a | 102.5 | 3370.99 ± 112.25 a | 3557.50 ± 209.34 a | 105.5 |
| | Aspartic acid | Pulp | 4460.50 ± 204.76 a | 2344.28 ± 175.58 b | 52.6 | 5372.43 ± 220.80 a | 3441.42 ± 188.07 b | 64.1 |
| | Glutamic acid | Pulp | 1204.90 ± 129.47 a | 669.90 ± 88.80 b | 55.6 | 1382.84 ± 27.66 a | 859.02 ± 70.09 b | 62.1 |
| | Serine | Pulp | 1160.02 ± 62.22 a | 690.58 ± 53.96 b | 59.5 | 1044.73 ± 14.92 a | 725.31 ± 44.27 b | 69.4 |
| | Alanine | Pulp | 1070.60 ± 58.27 a | 745.25 ± 148.67 a | 69.6 | 882.06 ± 49.38 a | 744.31 ± 18.96 b | 84.3 |
| | Glycine | Pulp | 124.22 ± 7.43 a | 71.68 ± 7.40 b | 57.7 | 103.03 ± 0.78 a | 69.98 ± 9.34 b | 67.9 |
| | Tyrosine | Pulp | 26.70 ± 1.21 a | 30.58 ± 3.95 a | 114.6 | 23.32 ± 0.80 b | 35.46 ± 4.93 a | 152.2 |
| | Cysteine | Pulp | ND | ND | - | ND | ND | - |
| | Total free amino acid | Pulp | 22809.89 ± 901.76 a | 17883.69 ± 1348.64 b | 78.1 | 20468.97 ± 225.92 a | 15866.84 ± 1054.54 b | 77.9 |
| Bitter Limonoids | Limonin | Pulp | 339.44±5.55 a | 338.09±9.98 a | 99.6 | 267.93±21.09 a | 328.65±84.66 a | 122.7 |
| | Limonin | Flav | 339.99±106.87 b | 736.89±109.89 a | 216.7 | 72.60±5.44 b | 414.55±110.25 a | 571.0 |
| | Limonin | Alb | 145.37±16.92 b | 922.82±200.33 a | 634.8 | 89.66±31.05 b | 508.98±163.17 a | 567.7 |

| | | | | | | | | |
|---|---|---|---|---|---|---|---|---|
| | Limonin | Seg | 1210.53±73.40 a | 1586.67±188.77 a | 131.1 | 1177.21±119.01 a | 1399.62±236.41 a | 118.9 |
| | Limonin | Sds | 8437.65±208.01 b | 9058.46±73.67 a | 107.4 | 8751.99±161.72 a | 8930.43±147.22 a | 102.0 |
| | Nomilin | Pulp | 20.67±0.02 a | 13.85±6.35 a | 67.0 | 11.33±0.76 a | 16.14±7.21 a | 142.5 |
| | Nomilin | Flav | 115.48±16.41 a | 99.94±6.31 a | 86.5 | 123.18±6.61 a | 108.45±1.81 a | 88.0 |
| | Nomilin | Alb | 7.77±2.24 a | 14.27±7.48 a | 183.7 | 12.34±0.79 a | 9.79±3.20 a | 79.3 |
| | Nomilin | Seg | 95.27±68.30 a | 113.81±62.71 a | 119.5 | 71.52±27.82 a | 91.25±24.59 a | 127.6 |
| | Nomilin | Sds | 2444.77±20.43 a | 1603.96±7.80 b | 65.6 | 1434.40±35.07 a | 1401.11±16.89 a | 97.7 |
| Flavonoids | Tangeretin | Pulp | 1.89±1.08 a | 1.69±0.44 a | 89.4 | 2.39±0.71 a | 2.30±0.95 a | 96.2 |
| | Tangeretin | Flav | 546.68±78.42 a | 519.88±57.83 a | 95.1 | 564.26±23.47 a | 518.44±18.76 b | 91.9 |
| | Tangeretin | Alb | 45.56±15.88 a | 40.51±15.14 a | 88.9 | 81.17±6.19 a | 58.15±4.90 b | 71.6 |
| | Tangeretin | Seg | 6.25±2.20 a | 6.21±1.97 a | 99.4 | 10.06±2.13 a | 5.93±0.95 b | 58.9 |
| | Tangeretin | Sds | 1.79±0.45 a | 4.56±4.58 a | 254.7 | 3.44±0.47 a | 2.87±3.00 a | 83.4 |
| | Sinensetin | Pulp | 0.135±0.060 a | 0.0000±0.0000 b | 0.0 | 0.043±0.019 a | 0.0000±0.0000 b | 0.0 |
| | Sinensetin | Flav | 18.39±1.49 a | 0.0000±0.0000 b | 0.0 | 6.37±0.47 a | 0.0000±0.0000 b | 0.0 |
| | Sinensetin | Alb | 1.69±0.50 a | 0.0000±0.0000 b | 0.0 | 1.10±0.19 a | 0.0000±0.0000 b | 0.0 |
| | Sinensetin | Seg | 0.250±0.095 a | 0.0000±0.0000 b | 0.0 | 0.182±0.065 a | 0.0000±0.0000 b | 0.0 |
| | Sinensetin | Sds | 0.085±0.005 a | 0.0000±0.0000 b | 0.0 | 0.105±0.005 a | 0.0000±0.0000 b | 0.0 |
| | Rhoifolin | Pulp | 9.83±1.00 a | 10.49±0.78 a | 106.7 | 6.07±0.93 b | 7.71±0.67 a | 127.0 |
| | Rhoifolin | Flav | 366.58±105.02 a | 305.93±31.36 a | 83.5 | 174.70±47.32 a | 151.40±18.02 a | 86.7 |
| | Rhoifolin | Alb | 484.31±35.82 a | 455.15±19.60 a | 94.0 | 341.55±109.90 a | 277.05±77.70 a | 81.1 |
| | Rhoifolin | Seg | 15.19±0.59 a | 15.68±1.68 a | 103.2 | 11.13±3.36 a | 8.96±1.64 a | 80.5 |
| | Rhoifolin | Sds | 2.85±0.32 a | 4.74±5.21 a | 166.3 | 3.91±0.43 a | 3.06±3.40 a | 78.3 |
| | Nobiletin | Pulp | 3.60±1.46 a | 4.83±1.46 a | 134.2 | 4.18±0.72 a | 4.86±1.51 a | 116.3 |
| | Nobiletin | Flav | 1629.92±175.77 b | 1970.86±150.82 a | 120.9 | 1364.55±34.58 b | 1565.16±50.89 a | 114.7 |
| | Nobiletin | Alb | 114.14±34.51 a | 103.93±32.92 a | 91.1 | 175.47±18.80 a | 133.85±10.07 b | 76.3 |
| | Nobiletin | Seg | 17.04±6.21 a | 17.26±6.56 a | 101.3 | 22.87±4.87 a | 13.92±2.36 b | 60.9 |
| | Nobiletin | Sds | 3.56±0.66 a | 5.52±3.75 a | 155.1 | 6.55±0.59 a | 3.11±2.48 b | 47.5 |
| | Didymin | Pulp | 9.50±0.46 a | 8.14±0.08 b | 85.7 | 5.33±0.15 a | 6.31±1.47 a | 118.4 |
| | Didymin | Flav | 13.38±5.39 a | 12.87±0.86 a | 96.2 | 10.35±7.30 a | 4.68±0.21 a | 45.2 |

| | | | | | | | |
|---|---|---|---|---|---|---|---|
| Didymin | Alb | 163.86±9.20 a | 172.17±15.29 a | 105.1 | 125.82±10.05 a | 115.98±22.89 a | 92.2 |
| Didymin | Seg | 115.87±24.42 a | 125.22±25.36 a | 108.1 | 94.23±10.43 a | 81.22±16.57 a | 86.2 |
| Didymin | Sds | 0.775±0.097 a | 4.15±5.05 a | 535.5 | 1.02±0.10 a | 3.37±4.56 a | 330.4 |
| Poncirin | Pulp | 10.79±0.60 a | 8.27±0.10 b | 76.6 | 5.97±0.24 a | 6.84±1.59 a | 114.6 |
| Poncirin | Flav | 14.61±6.01 a | 11.72±0.66 a | 80.2 | 11.85±7.96 a | 4.66±0.28 a | 39.3 |
| Poncirin | Alb | 174.31±8.46 a | 155.03±13.82 b | 88.9 | 139.06±12.38 a | 110.69±16.45 b | 79.6 |
| Poncirin | Seg | 127.21±26.22 a | 117.61±21.50 a | 92.5 | 103.25±10.67 a | 80.80±14.80 b | 78.3 |
| Poncirin | Sds | 0.855±0.046 a | 5.85±5.82 a | 684.2 | 1.11±0.09 a | 4.01±4.89 a | 361.3 |
| Neohesperidin | Pulp | 803.58±81.50 a | 568.80±80.46 b | 70.8 | 689.88±20.33 a | 510.56±76.18 b | 74.0 |
| Neohesperidin | Flav | 3670.97±737.84 a | 2968.26±211.42 a | 80.9 | 2520.11±653.60 a | 1466.01±121.46 b | 58.2 |
| Neohesperidin | Alb | 8610.68±273.66 a | 8897.25±209.69 a | 103.3 | 7961.02±422.59 a | 7093.93±311.68 b | 89.1 |
| Neohesperidin | Seg | 4380.34±406.20 a | 4134.32±538.16 a | 94.4 | 3911.98±353.23 a | 3011.85±399.38 b | 77.0 |
| Neohesperidin | Sds | 45.84±1.92 a | 24.00±10.93 b | 52.4 | 77.18±7.96 a | 13.25±4.87 b | 17.2 |
| Narirutin | Pulp | 1914.77±138.92 a | 1456.22±30.94 b | 76.1 | 2041.32±226.02 a | 1636.00±75.47 b | 80.1 |
| Narirutin | Flav | 2337.68±205.93 a | 1934.41±257.45 b | 82.7 | 2170.08±351.93 a | 1426.06±61.90 b | 65.7 |
| Narirutin | Alb | 7208.49±363.30 a | 7077.64±471.62 a | 98.2 | 6851.96±268.01 a | 5911.63±269.55 b | 86.3 |
| Narirutin | Seg | 5718.76±130.25 a | 5417.75±321.80 a | 94.7 | 5962.30±255.74 a | 4784.09±354.71 b | 80.2 |
| Narirutin | Sds | 117.04±3.37 a | 58.48±37.68 b | 50.0 | 142.66±10.41 a | 42.81±25.76 b | 30.0 |
| Naringin | Pulp | 2336.05±222.94 a | 1750.94±33.01 b | 75.0 | 2297.51±216.30 a | 1851.04±48.72 b | 80.6 |
| Naringin | Flav | 4400.36±457.91 a | 3668.52±456.98 b | 83.4 | 3988.16±694.95 a | 2562.26±92.56 b | 64.2 |
| Naringin | Alb | 13580.56±721.29 a | 13680.94±776.56 a | 100.7 | 12686.32±457.65 a | 11321.75±478.25 b | 89.2 |
| Naringin | Seg | 9444.41±282.95 a | 9206.24±622.41 a | 97.5 | 9596.30±649.85 a | 7782.60±698.50 b | 81.1 |
| Naringin | Sds | 144.01±3.13 a | 65.78±43.28 b | 45.7 | 191.50±15.66 a | 46.37±29.02 b | 24.2 |
| Hesperidin | Pulp | 624.24±53.38 a | 441.40±60.33 b | 70.7 | 557.32±25.38 a | 413.54±60.11 b | 74.2 |
| Hesperidin | Flav | 2628.28±525.96 a | 2084.44±133.99 a | 79.3 | 1789.25±476.17 a | 1021.25±94.16 b | 57.1 |
| Hesperidin | Alb | 6301.70±203.39 a | 6315.79±120.11 a | 100.2 | 5884.00±358.39 a | 5024.20±187.62 b | 85.4 |
| Hesperidin | Seg | 3219.72±314.29 a | 2975.32±354.14 a | 92.4 | 2844.28±237.89 a | 2177.91±285.26 b | 76.6 |
| Hesperidin | Sds | 35.59±2.18 a | 21.85±9.14 b | 61.4 | 59.78±5.60 a | 13.63±3.64 b | 22.8 |
| Hesperetin | Pulp | n.d. | 0.494±0.163 a | - | n.d. | 0.347±0.040 a | - |

| | | | | | | | |
|---|---|---|---|---|---|---|---|
| Hesperetin | Flav | 0.655±0.189 b | 1.77±1.03 a | 270.2 | 0.518±0.190 b | 1.09±0.25 a | 210.4 |
| Hesperetin | Alb | 0.788±0.095 a | 0.676±0.062 b | 85.8 | 1.86±1.66 a | 0.848±0.060 a | 45.6 |
| Hesperetin | Seg | 0.253±0.048 b | 0.531±0.096 a | 209.9 | 0.470±0.210 a | 0.432±0.068 a | 91.9 |
| Hesperetin | Sds | 0.125±0.023 a | 3.59±5.46 a | 2872.0 | 0.107±0.019 a | 4.63±5.28 a | 4327.1 |
| Naringenin | Pulp | n.d. | 1.45±0.21 a | - | n.d. | 1.31±0.14 a | - |
| Naringenin | Flav | 0.902±0.279 a | 1.54±0.64 a | 170.7 | 1.08±0.86 a | 1.14±0.32 a | 105.6 |
| Naringenin | Alb | 1.23±0.16 a | 1.21±0.02 a | 98.4 | 6.59±8.14 a | 1.40±0.18 a | 21.2 |
| Naringenin | Seg | 0.588±0.170 b | 1.38±0.24 a | 234.7 | 1.65±1.54 a | 1.43±0.33 a | 86.7 |
| Naringenin | Sds | 1.94±0.07 a | 4.00±4.99 a | 206.2 | 1.10±0.09 a | 3.53±4.84 a | 320.9 |
| Rutin | Pulp | 655.83±62.34 a | 480.90±74.17 b | 73.3 | 580.80±20.77 a | 445.11±63.41 b | 76.6 |
| Rutin | Flav | 2857.17±608.83 a | 2342.80±150.82 a | 82.0 | 1933.26±502.47 a | 1154.76±108.93 b | 59.7 |
| Rutin | Alb | 6893.90±246.18 a | 6998.70±149.08 a | 101.5 | 6451.03±435.98 a | 5637.27±211.20 b | 87.4 |
| Rutin | Seg | 3493.64±346.07 a | 3343.98±430.14 a | 95.7 | 3081.73±241.75 a | 2410.74±315.59 b | 78.2 |
| Rutin | Sds | 35.81±2.04 a | 22.63±9.92 b | 63.2 | 60.91±5.87 a | 13.89±3.63 b | 22.8 |
| Eriocitrin | Pulp | 706.59±37.77 a | 514.11±69.02 b | 72.8 | 767.71±82.05 a | 483.56±19.52 b | 63.0 |
| Eriocitrin | Flav | 517.30±26.44 a | 351.21±73.66 b | 67.9 | 574.35±95.48 a | 316.32±21.72 b | 55.1 |
| Eriocitrin | Alb | 820.77±138.91 a | 624.72±80.45 b | 76.1 | 1072.23±78.61 a | 653.24±22.12 b | 60.9 |
| Eriocitrin | Seg | 1012.90±34.52 a | 730.13±33.88 b | 72.1 | 1368.91±73.66 a | 717.70±43.91 b | 52.4 |
| Eriocitrin | Sds | 26.58±0.97 a | 15.99±4.99 b | 60.2 | 42.16±4.27 a | 14.45±4.07 b | 34.3 |
| Neoeriocitrin | Pulp | 377.79±52.63 b | 478.32±62.98 a | 126.6 | 358.76±16.43 b | 447.52±24.12 a | 124.7 |
| Neoeriocitrin | Flav | 3057.36±395.38 a | 360.11±73.48 b | 11.8 | 2981.51±317.16 a | 308.36±20.55 b | 10.3 |
| Neoeriocitrin | Alb | 4002.37±248.80 a | 677.34±109.13 b | 16.9 | 4084.74±249.38 a | 647.44±32.43 b | 15.9 |
| Neoeriocitrin | Seg | 1942.04±147.61 a | 780.42±51.80 b | 40.2 | 2099.08±233.58 a | 734.29±44.96 b | 35.0 |
| Neoeriocitrin | Sds | 22.94±1.64 a | 20.78±8.63 a | 90.6 | 37.44±2.91 a | 19.61±8.20 b | 52.4 |
| Total Flavonoids | Pulp | 7454.60±294.25 a | 5726.06±368.88 a | 76.8 | 7317.28±326.13 a | 5817.01±224.91 a | 79.5 |
| Total Flavonoids | Flav | 22060.24±1284.12 a | 16534.31±1168.85 a | 75.0 | 18090.40±1275.55 a | 10501.59±281.38 a | 58.1 |
| Total Flavonoids | Alb | 48404.36±955.61 a | 45201.06±1924.90 a | 93.4 | 45863.92±927.11 a | 36987.43±1467.44 a | 80.6 |

| | | | | | | | |
|---|---|---|---|---|---|---|---|
| | Total Flavonoids | Seg | 29494.46±710.55 a | 26872.05±2327.02 a | 91.1 | 29108.42±887.56 a | 21811.87±2140.53 a | 74.9 |
| | Total Flavonoids | Sds | 439.79±6.18 a | 259.97±89.33 b | 59.1 | 628.97±22.59 a | 186.38±49.22 b | 29.6 |
| Phenolic Acids | Protocatechuic acid | Pulp | 4.50±0.25 b | 5.51±0.25 a | 122.4 | 4.66±0.23 a | 3.71±1.52 a | 79.6 |
| | Protocatechuic acid | Flav | 19.17±0.83 a | 18.75±2.69 a | 97.8 | 17.79±1.41 a | 13.97±2.11 b | 78.5 |
| | Protocatechuic acid | Alb | 6.10±0.29 a | 5.32±0.24 b | 87.2 | 5.73±0.70 a | 8.64±4.53 a | 150.8 |
| | Protocatechuic acid | Seg | 4.08±0.23 a | 3.62±0.20 b | 88.7 | 3.03±0.27 a | 3.42±0.25 a | 112.9 |
| | Protocatechuic acid | Sds | 5.29±0.09 a | n.d. | - | 5.52±0.04 a | n.d. | - |
| | p-Hydroxybenzoic acid | Pulp | 7.50±0.45 a | 7.67±0.12 a | 102.3 | 6.91±0.13 a | 7.01±0.26 a | 101.4 |
| | p-Hydroxybenzoic acid | Flav | 58.76±0.21 a | 67.48±6.50 a | 114.8 | 56.21±4.61 a | 56.29±3.87 a | 100.1 |
| | p-Hydroxybenzoic acid | Alb | 85.01±6.60 a | 86.24±4.88 a | 101.4 | 76.82±4.91 a | 80.43±0.60 a | 104.7 |
| | p-Hydroxybenzoic acid | Seg | 31.82±0.45 a | 32.63±1.32 a | 102.5 | 28.57±1.02 a | 31.45±2.09 a | 110.1 |
| | p-Hydroxybenzoic acid | Sds | 9.20±0.02 a | n.d. | - | 10.70±0.02 a | n.d. | - |
| | Caffeic acid | Pulp | 23.85±0.42 a | 24.14±0.70 a | 101.2 | 22.58±0.80 a | 21.04±0.59 a | 93.2 |
| | Caffeic acid | Flav | 38.74±2.86 a | 42.24±3.46 a | 109.0 | 20.78±1.68 a | 16.68±0.66 b | 80.3 |
| | Caffeic acid | Alb | 61.69±10.75 a | 52.45±13.12 a | 85.0 | 36.29±14.38 a | 29.56±9.50 a | 81.5 |
| | Caffeic acid | Seg | 27.72±6.64 a | 24.35±3.55 a | 87.8 | 20.61±1.45 a | 19.48±1.76 a | 94.5 |
| | Caffeic acid | Sds | 21.51±0.28 a | n.d. | - | 24.19±0.10 a | n.d. | - |
| | Vanillic acid | Pulp | 35.95±2.10 a | 36.56±0.87 a | 101.7 | 35.34±0.17 a | 33.33±2.14 a | 94.3 |
| | Vanillic acid | Flav | 167.25±5.16 b | 191.29±9.60 a | 114.4 | 153.45±5.16 a | 152.89±6.09 a | 99.6 |
| | Vanillic acid | Alb | 162.48±2.57 a | 160.88±2.13 a | 99.0 | 155.70±6.26 a | 157.10±6.46 a | 100.9 |
| | Vanillic acid | Seg | 74.86±5.40 a | 70.79±6.74 a | 94.6 | 70.42±0.50 a | 72.45±1.97 a | 102.9 |
| | Vanillic acid | Sds | 28.26±0.77 a | n.d. | - | 30.41±0.43 a | n.d. | - |
| | p-Coumaric acid | Pulp | 14.98±0.45 a | 14.05±0.10 a | 93.8 | 15.32±0.88 a | 15.16±0.40 a | 99.0 |
| | p-Coumaric acid | Flav | 128.33±8.80 a | 146.58±2.02 a | 114.2 | 91.93±1.55 a | 98.03±6.75 a | 106.6 |

| | | | | | | | |
|---|---|---|---|---|---|---|---|
| p-Coumaric acid | Alb | 57.48±4.81 a | 57.01±6.91 a | 99.2 | 42.99±5.72 a | 41.94±2.54 a | 97.6 |
| p-Coumaric acid | Seg | 35.44±3.90 a | 34.82±3.04 a | 98.3 | 28.39±0.28 a | 28.71±1.29 a | 101.1 |
| p-Coumaric acid | Sds | 94.53±0.89 a | n.d. | - | 105.61±0.30 a | n.d. | - |
| Ferulic acid | Pulp | 173.20±4.37 a | 170.55±2.87 a | 98.5 | 182.94±3.30 a | 181.15±3.15 a | 99.0 |
| Ferulic acid | Flav | 1208.93±38.07 b | 1386.53±19.10 a | 114.7 | 976.59±18.49 a | 1044.58±53.86 a | 107.0 |
| Ferulic acid | Alb | 259.80±3.70 a | 243.85±9.88 b | 93.9 | 293.43±56.26 a | 273.07±30.10 a | 93.1 |
| Ferulic acid | Seg | 152.52±8.63 a | 165.11±13.52 a | 108.3 | 140.12±8.50 a | 143.62±6.39 a | 102.5 |
| Ferulic acid | Sds | 105.79±1.08 a | n.d. | - | 128.29±0.45 a | n.d. | - |
| Sinapic acid | Pulp | 61.46±1.04 a | 64.91±5.27 a | 105.6 | 56.40±1.25 a | 58.55±0.91 a | 103.8 |
| Sinapic acid | Flav | 349.38±21.07 a | 390.34±21.30 a | 111.7 | 329.50±25.51 a | 358.45±42.53 a | 108.8 |
| Sinapic acid | Alb | 110.25±5.99 a | 112.88±6.84 a | 102.4 | 126.78±17.63 a | 115.94±16.16 a | 91.4 |
| Sinapic acid | Seg | 91.80±1.75 b | 104.35±0.93 a | 113.7 | 83.03±12.97 a | 107.28±1.68 a | 129.2 |
| Sinapic acid | Sds | 17.40±0.09 a | n.d. | - | 23.43±0.01 a | n.d. | - |
| Total Phenolic Acids | Pulp | 321.44±2.25 b | 646.77±20.04 a | 201.2 | 324.15±2.16 b | 639.91±3.79 a | 197.4 |
| Total Phenolic Acids | Flav | 1970.58±34.41 b | 4486.38±135.07 a | 227.7 | 1646.24±18.63 a | 3481.80±272.77 a | 211.5 |
| Total Phenolic Acids | Alb | 742.81±1.95 b | 1437.25±49.69 a | 193.5 | 737.75±105.76 b | 1413.39±148.81 a | 191.6 |
| Total Phenolic Acids | Seg | 418.24±26.28 b | 871.31±69.62 a | 208.3 | 374.17±2.39 b | 812.80±28.44 a | 217.2 |
| Total Phenolic Acids | Sds | 281.97±3.01 a | n.d. | - | 328.14±0.68 a | n.d. | - |

**Table S10. Effect of drying methods on total flavonoids, total phenolic acids, FRAP, DPPH and ABTS in Huyou fruits.**

| Metabolite Class | Compound | Tissue | HY-N1 Freeze-dried | HY-N1 Hot-air dried | HY-N1 Ret. (%) | HY-17 Freeze-dried | HY-17 Hot-air dried | HY-17 Ret. (%) |
|---|---|---|---|---|---|---|---|---|
| | Total Flavonoids | Pulp | 1.65±0.10 a | 1.87±0.11 a | 113.3 | 1.65±0.03 b | 1.93±0.07 a | 117.0 |
| | Total Flavonoids | Flav | 6.88±0.51 a | 7.12±0.61 a | 103.5 | 6.15±0.75 a | 6.02±0.31 a | 97.9 |
| | Total Flavonoids | Alb | 11.13±0.19 a | 10.80±0.44 a | 97.0 | 13.63±5.61 a | 9.58±0.45 a | 70.3 |
| | Total Flavonoids | Seg | 6.12±0.42 a | 6.31±0.30 a | 103.1 | 5.81±0.27 a | 5.68±0.50 a | 97.8 |
| | Total Flavonoids | Sds | 1.45±0.02 a | 1.43±0.04 a | 98.6 | 1.64±0.01 a | 1.56±0.10 a | 95.1 |
| | Total Phenolic Acids | Pulp | 7.30±0.21 b | 8.98±0.48 a | 123.0 | 6.78±0.10 b | 8.99±0.53 a | 132.6 |
| | Total Phenolic Acids | Flav | 20.05±0.99 b | 22.71±0.75 a | 113.3 | 18.55±2.41 a | 19.15±0.72 a | 103.2 |
| | Total Phenolic Acids | Alb | 28.72±0.39 a | 28.70±1.71 a | 99.9 | 28.96±9.13 a | 24.98±1.73 a | 86.3 |
| | Total Phenolic Acids | Seg | 15.68±0.61 a | 15.95±0.55 a | 101.7 | 14.87±1.03 a | 13.95±1.00 a | 93.8 |
| | Total Phenolic Acids | Sds | 2.34±0.02 a | 2.32±0.04 a | 99.1 | 2.32±0.02 a | 2.36±0.22 a | 101.7 |
| Total Antioxidant Capacity | FRAP (μmol Trolox/g) | Pulp | 19.79±0.24 a | 8.37±0.21 b | 42.3 | 7.62±1.77 a | 8.23±0.74 a | 108.0 |
| Total Antioxidant Capacity | FRAP (μmol Trolox/g) | Flav | 20.67±2.00 b | 31.98±4.57 a | 154.7 | 13.60±2.33 b | 35.07±7.37 a | 257.9 |
| Total Antioxidant Capacity | FRAP (μmol Trolox/g) | Alb | 16.12±1.54 a | 17.53±2.56 a | 108.7 | 29.73±6.12 a | 17.22±0.30 b | 57.9 |
| Total Antioxidant Capacity | FRAP (μmol Trolox/g) | Seg | 15.13±0.28 a | 10.79±3.49 a | 71.3 | 9.75±0.91 b | 16.85±1.41 a | 172.8 |
| Total Antioxidant Capacity | FRAP (μmol Trolox/g) | Sds | 4.15±0.32 a | 3.62±0.02 a | 87.2 | 4.00±0.19 a | 4.47±0.47 a | 111.8 |
| Total Antioxidant Capacity | DPPH (μmol Trolox/g) | Pulp | 33.97±3.93 a | 30.31±0.31 a | 89.2 | 27.51±2.71 a | 30.66±3.50 a | 111.5 |
| Total | DPPH (μmol Trolox/g) | Flav | 56.27±3.73 b | 79.14±7.38 a | 140.6 | 53.98±2.81 b | 100.51±6.93 a | 186.2 |

| | | | | | | | | |
|---|---|---|---|---|---|---|---|---|
| Antioxidant Capacity | | | | | | | | |
| Total Antioxidant Capacity | DPPH (µmol Trolox/g) | Alb | 52.73±0.33 a | 50.05±14.91 a | 94.9 | 74.92±18.61 a | 56.53±2.73 a | 75.5 |
| Total Antioxidant Capacity | DPPH (µmol Trolox/g) | Seg | 32.18±7.20 a | 34.42±4.60 a | 107.0 | 47.45±6.20 a | 33.52±4.62 b | 70.6 |
| Total Antioxidant Capacity | DPPH (µmol Trolox/g) | Sds | 7.29±0.00 a | 5.32±0.90 a | 73.0 | 5.66±0.00 a | 8.62±0.34 a | 152.3 |
| Total Antioxidant Capacity | ABTS (µmol Trolox/g) | Pulp | 39.14±0.71 b | 49.60±3.42 a | 126.7 | 36.72±3.06 a | 47.70±5.52 a | 129.9 |
| Total Antioxidant Capacity | ABTS (µmol Trolox/g) | Flav | 81.86±4.10 a | 80.53±7.92 a | 98.4 | 68.53±18.50 a | 85.62±5.33 a | 124.9 |
| Total Antioxidant Capacity | ABTS (µmol Trolox/g) | Alb | 122.14±10.64 a | 128.62±7.25 a | 105.3 | 151.27±68.76 a | 118.35±12.45 a | 78.2 |
| Total Antioxidant Capacity | ABTS (µmol Trolox/g) | Seg | 77.35±4.89 a | 79.00±0.97 a | 102.1 | 73.57±3.73 a | 79.06±0.54 a | 107.5 |
| Total Antioxidant Capacity | ABTS (µmol Trolox/g) | Sds | 15.15±0.00 a | 13.52±0.76 a | 89.2 | 13.87±0.00 a | 14.69±0.21 a | 105.9 |
