# Supplementary material for: Tissue-Specific Phytochemical Profiles of Two Huyou Cultivars: Bioactive Compounds and Thermal Processing Effects
Source: Foods. 2026 Oct 23;15(21):3805. doi: 10.3390/foods15213805 (PMC13762711; doi:10.3390/foods15213805)
Supplement: Supplementary file 1 [file foods-15-03805-s001.zip › Figure S1.pdf]

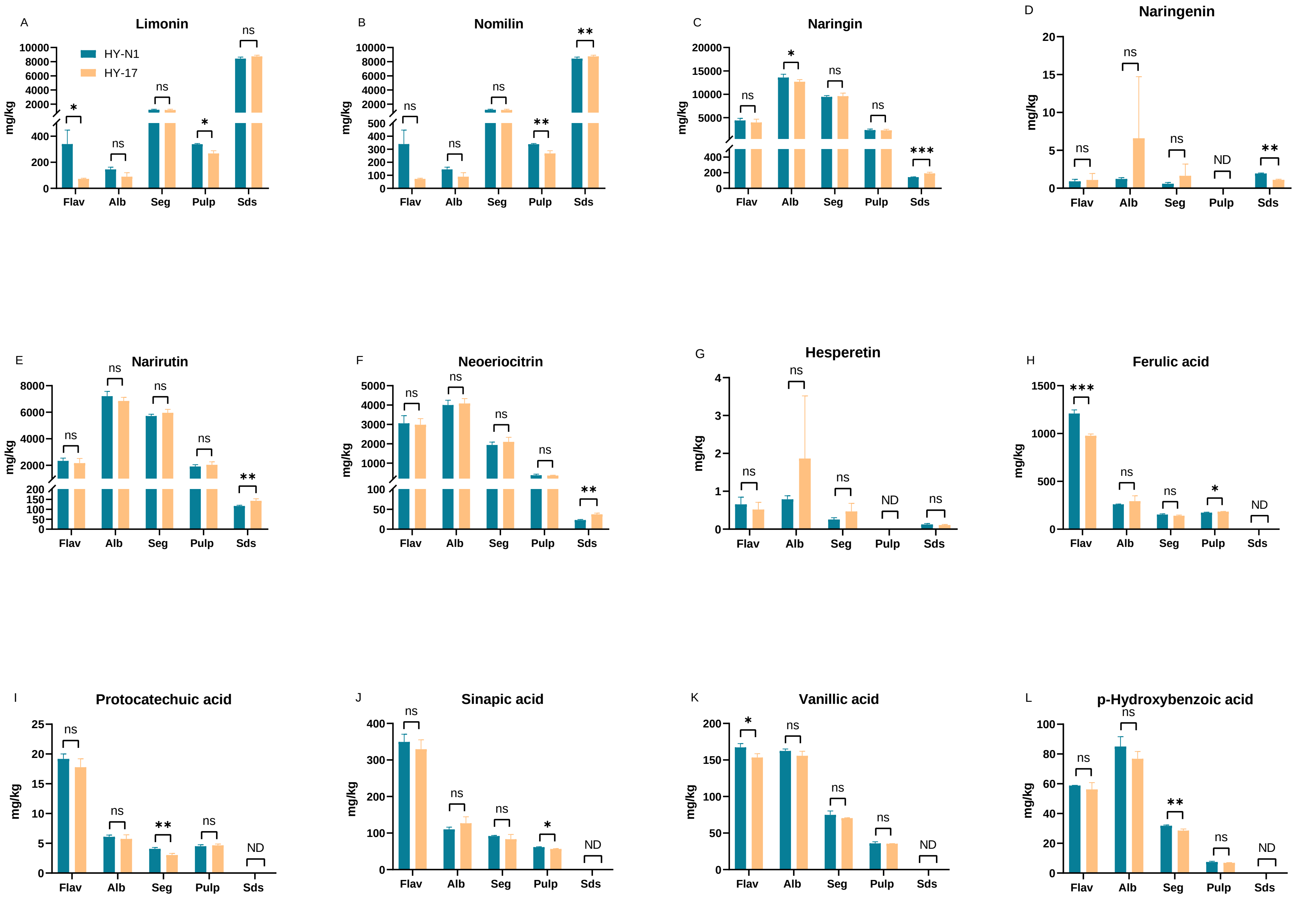

A
Limonin
HY-N1
HY-17
B
Nomilin
C
Naringin
D
Naringenin
E
Narirutin
F
Neoeriocitrin
G
Hesperetin
H
Ferulic acid
I
Protocatechuic acid
J
Sinapic acid
K
Vanillic acid
L
p-Hydroxybenzoic acid
mg/kg
Flav
Alb
Seg
Pulp
Sds
ns
ND
